# An optimized three-laser 27-color spectral flow cytometry panel for multi-organ profiling in mice

**DOI:** 10.1371/journal.pone.0347810

**Published:** 2026-07-20

**Authors:** Hyeonji Song, Yusik Lim, Jaechul Lim

**Affiliations:** 1 College of Veterinary Medicine, Seoul National University, Seoul, Republic of Korea; 2 LK Bioscience Inc., Kyunggi-do, Republic of Korea; 3 The Research Institute for Veterinary Science, Seoul National University, Seoul, Republic of Korea; Indian Institute of Chemical Technology, INDIA

## Abstract

While high-dimensional flow cytometry plays critical roles in resolving complex cellular networks, there remains a scarcity of comprehensive panels for the simultaneous profiling of diverse mouse cell types, primarily due to the technical challenges of spectral overlap and the limited availability of mouse-validated reagents. To address this technical gap and resolve diverse cell populations in murine models, we designed a 27-color flow cytometry panel optimized for 3-laser spectral flow cytometers. This optimized panel enables broad and simultaneous detection of 16 distinct cell subsets from both lymphoid and myeloid lineages—including T cells, B cells, plasma cells, NK cells, innate lymphoid cells, dendritic cells, monocytes, macrophages, neutrophils, eosinophils, basophils, mast cells—along with non-immune cells, such as epithelial, endothelial, fibroblast, and neuronal-associated cells. The panel has been successfully applied to various tissues, including spleen, thymus, bone marrow, peripheral blood, mesenteric lymph nodes, peritoneal lavage fluid, gut epithelium, and lamina propria. Applying this panel to a poly(I:C) model, we successfully tracked systemic shifts in monocyte and neutrophil populations and identified a previously unrecognized, *Cyp11b1*-expressing immune cell subset via reporter expression. This panel will facilitate high-dimensional immune profiling on standard 3-laser cytometers, providing a robust tool for dissecting cellular landscapes across diverse contexts.

## 1. Introduction

Mouse models are a cornerstone of modern immunology, offering unparalleled opportunities to investigate immune mechanisms at the cellular and organismal level [1]. Unlike most human studies that rely primarily on peripheral blood, murine systems readily allow direct analysis of immune responses within organs [2], thereby providing insight into tissue-resident and microenvironment-dependent immune regulation. Despite this advantage, most high-dimensional flow cytometry panels developed to date have concentrated on human samples, such as human PBMCs [3,4], T cells, or antigen-presenting cells [5,6]. Consequently, there is a critical need for high-dimensional panels capable of capturing a broader range of murine immune cell populations.

Notably, tissue-level immune analysis is particularly important owing to substantial heterogeneity in immune composition and activation states across lymphoid and non-lymphoid organs [7,8]. For example, mucosal tissues—such as the gut epithelium and lamina propria—are more complex than classical lymphoid organs, integrating inductive and tissue-resident compartments that balance tolerance and inflammation [9]. Despite their significance, these regions have been underrepresented in prior panel designs due to complex tissue digestion protocols and the resulting poor cell viability and low yield [10]. This need becomes even more apparent in systemic diseases such as sepsis, where dysregulated host responses extend across multiple organs and involve simultaneous alterations in diverse immune cell populations rather than a single dominant lineage [11]. In these settings, immune dysregulation is accompanied by organ-specific inflammatory and metabolic changes, as well as inter-organ crosstalk, such that peripheral blood measurements alone may not fully reflect the tissue-level immune programs contributing to disease progression and organ dysfunction [12,13]. Accordingly, deep cell-level profiling across anatomically distinct tissues is essential for resolving how complex immune states are distributed across organs and for identifying coordinated or divergent cellular responses in systemic disease.

## 2. Materials and methods

### 2.1. Animals

Wild-type C57BL/6J (RRID: IMSR_JAX:000664) and homozygous *Cyp11b1*^*mScarlet*^ reporter mice [14] were maintained under SPF conditions with food and water ad libitum. Female mice aged 6–8 weeks were used in all experiments. Wild-type mice were used for panel optimization, including antibody titration and control setup, whereas *Cyp11b1*^*mScarlet*^ reporter mice were used for biological application experiments. All procedures were approved by the Institutional Animal Care and Use Committee (IACUC) of Seoul National University (SNU-240705-4-1) and conformed to institutional and national guidelines. Experiments were conducted and reported in accordance with ARRIVE guidelines. At the experimental endpoint, mice were euthanized through the gradual introduction of carbon dioxide gas to minimize animal suffering and distress by inducing deep anesthesia. Death was subsequently confirmed via cardiac puncture for terminal blood collection prior to tissue harvesting.

### 2.2. Poly(I:C) treatment

*Cyp11b1*^*mScarlet*^ reporter mice were administered poly(I:C) (20 μg/g body weight; 2 mg/mL) via intraperitoneal injection (200 μL per mouse). Control *Cyp11b1*^*mScarlet*^ reporter mice received an equal volume of sterile saline. Tissues were collected 24 h after injection for flow cytometric analysis (*n = 4* mice per group; total *n = 8* mice).

### 2.3. Instrument configuration

The panel was designed for a 3 Laser Cytek® Aurora spectral flow cytometer with the laser and detector specification displayed here (S1 Table). Detector gains were applied according to the Cytek assay settings, with forward and side scatter voltages manually optimized to match the light scatter properties of the target cell populations.

### 2.4. Fluorochrome selection and marker assignment

For initial fluorochrome selection, candidates were guided by relative brightness indices and spectral overlap profiles to fit our specific 3-laser instrument availability. Following this selection, the structured framework for marker assignment was adapted from the supplemental strategy outlined in OMIP-069 [15].

First, markers were paired based on the inverse relationship between relative antigen density and fluorochrome brightness. Bright fluorochromes were assigned to low-abundance markers (RB744-CD117, PE-CD170), whereas dimmer fluorochromes were paired with highly expressed antigens (BV510-CD8, PerCP-Ly6G). Second, the Spillover Spreading Matrix (SSM) was evaluated to mitigate spreading risks. Fluorochrome combinations exhibiting a higher propensity for spreading error were selectively redistributed to mutually exclusive markers, preserving the resolution of co-expressing populations. Finally, these theoretical assignments were practically adjusted based on the commercial availability of specific antibody-fluorochrome conjugates. This entire optimization process, including spectral evaluations and matrix verifications, was conducted using Cytek’s Cloud Panel Builder (https://cloud.cytekbio.com), yielding the final validated panel framework (S1 Fig). Specifically, this process was enhanced by the platform’s artificial intelligence (AI)-driven SpectroPanel™ algorithm to manage instrument-specific spreading and similarity matrices within our manual design.

### 2.5. Reporter signal characterization

To incorporate the mScarlet reporter signal into the panel, the spectral profile of mScarlet was characterized using cells from *Cyp11b1*^*mScarlet*^ reporter mice. Its emission spectrum was compared with spectrally adjacent fluorochromes (PE, PE-eFluor 610, and PE-Fire 640) to evaluate potential overlap and confirm compatibility with the panel (S2 Fig). Additionally, full-panel stained wild-type (non-reporter) adrenal gland cells were analyzed in parallel to confirm the specificity of the mScarlet signal (S3 Fig). Based on this evaluation, mScarlet could be resolved as an additional parameter without unacceptable interference.

### 2.6. Spectral unmixing and reference controls

Spectral unmixing was performed using cell-based reference controls for each fluorochrome to ensure spectral identity with the experimental data. These controls were prepared using single-stained cell samples from the prior antibody titration phase, during which cells were harvested from tissues with robust marker expression and processed under standardized protocols to minimize biological variability. Although compensation beads provide high signal intensity, cell-based controls were preferentially utilized to prevent subtle spectral discrepancies between beads and biological cells, which can lead to cumulative unmixing errors in high-parameter panels [16,17].

While combining bead-based and cell-based controls within a single panel is an efficient alternative depending on marker availability, achieving a distinct positive population remains critical. In this study, all reference controls successfully met the minimum criteria for both negative and positive event counts required by the SpectroFlo Unmixing Wizard, ensuring adequate signal separation and reliable reference spectra.

### 2.7. Antibody titration

Antibody titrations were performed in a 50 μL total staining volume per well. For each antibody, defined amounts were added according to the titration design, starting at 1 μg per test and followed by two-fold serial dilutions down to 0.0625 μg (i.e., 1 μg, 0.5 μg, 0.25 μg, 0.125 μg, and 0.0625 μg). Based on the unmixing results, additional lower concentrations were tested for fluorochromes that exhibited excessive spillover or signal saturation. For these antibodies, 3–5 additional dilution steps below 0.0625 μg were evaluated to identify the concentration that provided optimal separation between positive and negative populations while minimizing spectral spreading (S4 Fig and S2 Table).

All antibody titrations were initially performed using splenocytes, which represent a broad range of hematopoietic populations. However, several markers showed low or inconsistent expression in the spleen, resulting in insufficient positive event counts. For these markers, alternative tissues known to express the target antigen—such as bone marrow for CD31, CD56, CD161, and CD170 or intestinal epithelium for CD326—were used for titration under identical staining and acquisition conditions. This approach ensured reliable determination of optimal antibody concentrations across all fluorochromes, supporting the generation of robust reference spectra in subsequent unmixing steps.

### 2.8. Staining protocol

Staining was performed as follows:

1) Spin down and resuspend with 200 µL of ice-cold PBS.
2) Perform LIVE/DEAD violet (Thermo Fisher Scientific, Cat# L34964): 50 µL/sample (0.0031 µL LIVE/DEAD violet in 49.69 µL PBS), 30 min at RT in the dark, add 150 µL PBS and spin down.
3) Wash with 200 µL PBS per well, spin down as before.
4) Perform FC block (RRID: AB_2922498) (1:500): 50 µL/sample, 15 min at 4°C in the dark, add 150 µL FACS buffer and spin down.
5) Wash with 200 µL FACS buffer per well, spin down as before.
6) Perform Monocyte block (BioLegend, Cat# 426102): 50 µL/sample (2.5 µL Monocyte Blocker in 47.5 µL FACS buffer), 15 min at 4°C in the dark, add 150 µL FACS buffer and spin down.
7) Wash with 200 µL FACS buffer per well, spin down as before.
8) Prepare the staining master mix by combining optimal concentrations from single-color titrations according to the pre-calculated template. Use Brilliant Stain Buffer (RRID: AB_2869750) as the base when two or more BV fluorochromes are included.
9) Perform surface staining: 50 µL/sample, 30 min at 4°C in the dark, add 150 µL FACS buffer and spin down.
10) Wash with 200 µL FACS buffer per well, spin down as before.
11) Filter cells through 40 µm nylon mesh and transfer to FACS tube.

### 2.9. Autofluorescence extraction for spectral unmixing

To determine the optimal unmixing model, unstained controls from each tissue were analyzed using the Multiple AF (autofluorescence) Explorer function in SpectroFlo. Although unstained cells from different tissues showed broadly similar AF profiles, subtle tissue-specific differences in signal intensity were observed across some detectors. In particular, tissues such as the gut exhibited elevated autofluorescence in specific channels. Unmixing without AF extraction left residual background signals, particularly in channels affecting population separation. Based on these comparisons, the Multiple AF Extraction (high AF) model was independently constructed and applied to each specific tissue using its corresponding unstained control, thereby precisely accommodating tissue-derived background variances (S5 Fig).

### 2.10. FMO controls for gate definition

Fluorescence minus one (FMO) controls were used for selected markers to support gate placement when positive and negative populations were not clearly separated after spectral unmixing. These controls were particularly useful for parameters affected by spectral spread and for defining gates for dim or infrequent populations with greater confidence (S6 Fig). Although FMOs were not generated for every marker, their targeted use helped improve gating consistency for selected populations.

### 2.11. Data analysis

All data were acquired on a 3-Laser Aurora system (Cytek Biosciences). Flow cytometry data were analyzed using FlowJo v10 (RRID: SCR_008520) and SpectroFlo v3.3.0 (RRID: SCR_025494). Dimensionality reduction was performed by UMAP (Uniform Manifold Approximation and Projection) using the FlowJo plugin, applied to gated cell populations from representative tissues. Statistical significance between two groups was assessed using the unpaired, non-parametric Mann–Whitney U test. Due to the exploratory nature of this multi-organ profiling study aimed at unbiased subset discovery, multiple-testing corrections were not applied to minimize Type II errors and avoid omitting potential biological discoveries. Instead, to mitigate the risk of false positives inherent to smaller sample sizes (*n = 4* per group), findings were interpreted by prioritizing highly conserved phenotypic trends and consistent directionality observed across multiple independent tissues.

## 3. Results

**Fig 1:**
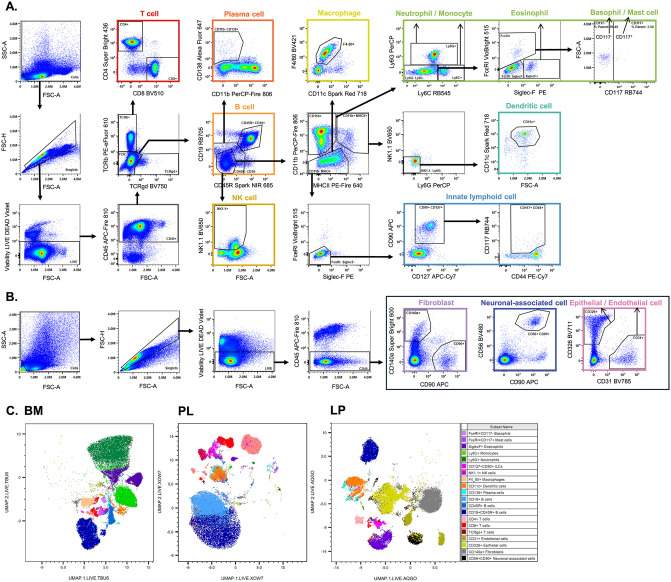
Gating Strategy. (A) Representative gating strategy for immune populations in the spleen. Populations were analyzed within the Cells/singlets/LIVE/CD45^+^ compartment and further resolved into major lymphoid and myeloid populations. (B) Representative gating strategy for non-immune populations in the small intestinal lamina propria. Populations were analyzed within the Cells/singlets/LIVE/CD45^-^ compartment to identify CD326^+^ epithelial cells, CD31^+^ endothelial cells, CD140a^+^ fibroblasts, and CD56^+^CD90^+^ neuronal-associated cells. (C) UMAP visualization of gated cell populations from bone marrow (left) and peritoneal lavage fluid (middle), and lamina propria (right). Cells were assigned to marker-defined populations based on the established gating strategy, and each color denotes a distinct annotated cell type shown in the legend.

To address the limited availability of broadly applicable high-dimensional flow cytometry panels for diverse murine tissues, we developed a 27-color spectral flow cytometry panel for comprehensive cellular profiling using a standard 3-laser configuration (Fig 1 and Table 1). Previously reported murine panels [18–28], have generally focused on lymphoid or myeloid subsets, or both only in limited settings, often within one or two organs and using four to five lasers. In contrast, our panel was designed to enable broad profiling across multiple murine tissues using a standard 3-laser spectral configuration. While standardized multicolor spectral frameworks utilizing a 3-laser configuration have already been established for human immunophenotyping [29], a corresponding 3-laser spectral panel remains absent for murine models. Even with the recent introduction of a high-dimensional murine spectral panel [27], such options strictly rely on 5-laser instrumentation and are tailored only to specific localized tissue environments, specifically the spleen and tumor. In contrast, our panel was strategically optimized through AI-assisted design to bridge this methodological gap, enabling robust 27-color profiling across eight distinct, independent murine tissues utilizing a widely accessible, standard 3-laser spectral configuration.

**Table 1:** Summary table for application of panel.

Purpose	Profiling of murine immune and non-immune cells in multiple organs
Species	Mouse
Cell type	Single-cell suspensions or leukocytes isolated from murine spleen, thymus, mesenteric lymph nodes, bone marrow, peripheral blood, peritoneal lavage, gut epithelium, and lamina propria
Cross references	OMIP-31, OMIP-32, OMIP-57, OMIP-59, OMIP-61, OMIP-76, OMIP-86, OMIP-93, OMIP-95, OMIP-104, OMIP-105

To construct the panel, markers were selected based on their expression patterns and immunological relevance, and fluorochromes were assigned with consideration of antigen density, signal resolution, and compatibility with spectral unmixing. Specifically, our panel is designed to simultaneously detect major lymphoid and myeloid lineages, using markers for T cells (CD4, CD8, TCRγδ), B cells (CD19, CD45R [B220]), plasma cell (CD138), NK cells (CD161 [NK1.1]), innate lymphoid cells (ILCs) (CD90 [Thy-1], CD127 [IL-7Rα], CD117 [c-Kit]), dendritic cells (CD11c), monocytes (Ly6C), macrophages (F4/80), neutrophils (Ly6G), eosinophils (CD170 [Siglec-F]), basophils, and mast cells (FcεRI, CD117 [c-Kit]), together with non-immune populations including endothelial cells (CD31 [PECAM-1]), epithelial cells (CD326 [EpCAM]), fibroblasts (CD140a [PDGFRα]), and neuronal-associated cells (CD56 [NCAM], CD90 [Thy-1]). To guide panel architecture, markers were grouped into three hierarchical categories—primary, secondary, and tertiary—according to their roles in major lineage identification, subset discrimination, and phenotypic refinement (S3 Table). Adapted from established immunophenotyping strategies, this framework provided a structured basis for panel design and interpretation of the cellular populations resolved by the panel while remaining compatible with spectral cytometry [6,30].

Having established the panel architecture, we next optimized the panel to improve signal resolution and unmixing accuracy under full multi-color conditions. Specifically, antibody concentrations for selected markers were slightly adjusted, and single-stain reference files were refined prior to performance assessment. Because spectral overlap among fluorochromes inherently introduces spreading that can compromise signal resolution, we then evaluated the impact of spectral spread by comparing reference controls with multi-color spleen samples (S7 Fig). This comparison revealed that signal separation was noticeably reduced in the multi-color samples, particularly among fluorochromes with highly overlapping emission spectra, demonstrating the inherent resolution loss caused by cumulative spectral spreading in multi-color conditions. Unmixing performance was evaluated using NxN matrix plots across all fluorochrome combinations, and manual adjustments were applied when residual spillover was detected (S8 Fig). Across all fluorochrome combinations examined, correction values remained below 5%, indicating overall stable unmixing performance across tissues. The most prominent example was the APC–Alexa Fluor 647 pair, in which correction values reached up to 4.96% in epithelial samples and exceeded 2% in several tissues, including bone marrow, peripheral leukocytes, and lamina propria (S9 Fig). Similar but less pronounced patterns were observed for a subset of additional fluorochromes. In the case of CD90 APC, the recurrent deviation from Alexa Fluor 647 likely reflected a reference control that was not as bright as the corresponding sample signal, which is a known source of reduced unmixing accuracy. Despite these localized deviations, overall signal separation remained sufficient to reliably resolve the targeted cell populations across tissues. Therefore, this panel offers an integrated view of the murine cellular landscape within a single experiment.

Notably, it was initially developed as a 26-color antibody panel, but its configuration does not spectrally overlap with the endogenous mScarlet fluorescent reporter [14], allowing the inclusion of this fluorescent protein as an additional independent parameter, resulting in a 27-color panel. This design ensures broad compatibility across immune-focused mouse models while maintaining high dimensionality within a compact 3-laser setup. Detailed information on the markers and fluorophores is provided in Table 2. Taken together, these features provide a foundation for tissue-resolved cellular analysis in mice.

**Table 2:** Antibodies used in the 27-color panel.

Specificity	Fluorochrome	Clone	Company	Catalog no.	Dilution	Peak channel
CD45	APC-Fire 810	30-F11	BioLegend	103174	1:641	R8
TCRβ	PE-eFluor 610	H57-597	Thermo Fisher Scientific	61-5961-82	1:160	B6
TCRγδ	BV750	GL3	BD Biosciences – OptiBuild	746962	1:80	V14
CD4	Super Bright 436	RM4–5	Cytek Biosciences	62-0042-82	1:80	V2
CD8	BV510	53-6.7	BioLegend	100752	1:160	V7
CD127 (IL-7Rα)	APC-Cy7	A7R34	BioLegend	135040	1:40	R7
CD19	RB705	1D3	BD Biosciences	570563	1:641	B10
CD45R (B220)	Spark NIR 685	RA3-6B2	BioLegend	103268	1:160	R3
CD138	Alexa Fluor 647	281−2	BioLegend	142526	1:100	R2
CD44	PE-Cy7	IM7	Cytek Biosciences	60-0441-U100	1:641	B13
MHC Class II (I-A + I-E)	PE-Fire 640	M5/114.15.2	BioLegend	107664	1:320	B7
CD161 (NK1.1)	BV650	PK136	Thermo Fisher Scientific	416-5941-82	1:80	V11
CD11b	PerCP-Fire 806	M1/70	BioLegend	101294	1:320	B14
CD11c	Spark Red 718	N418	BioLegend	117372	1:80	R4
F4/80	BV421	BM8	Thermo Fisher Scientific	404-4801-82	1:320	V1
FcεRI	Vio Bright B515	REA1079	Miltenyi Biotec	130-118-845	1:120	B1
CD117 (c-kit)	RB744	2B8	BD Biosciences	570552	1:320	B12
Ly6C	RB545	AL-21	BD Biosciences	569288	1:160	B3
Ly6G	PerCP	1A8	BioLegend	127654	1:80	B8
CD170 (Siglec-F)	PE	1RNM44N	Thermo Fisher Scientific	12-1702	1:160	B4
CD31 (PECAM-1)	BV785	390	BioLegend	102435	1:320	V15
CD326 (Ep-CAM)	BV711	G8.8	Thermo Fisher Scientific	407-5791-82	1:80	V13
CD56 (NCAM)	BV480	809220	BD Biosciences – OptiBuild	748095	1:80	V5
CD90 (Thy-1)	APC	G7	Thermo Fisher Scientific	A14727	1:641	R1
CD140a (PDGFRα)	Super Bright 600	APA5	Thermo Fisher Scientific	63-1401-82	1:80	V10
Viability	LIVE DEAD Violet	–	Thermo Fisher Scientific	L34964	1:16,000	V3
–	mScarlet	–	–	–	–	B5

To validate its utility, we next applied this panel to resolve major immune and non-immune cell populations across multiple murine tissues, including spleen, thymus, mesenteric lymph nodes, bone marrow, peripheral blood leukocytes, peritoneal lavage fluid, gut epithelium, and lamina propria, using a unified hierarchical gating framework. While the overarching gating logic was conserved across all organs, the precise gating boundaries were empirically adjusted for each tissue to account for microenvironmental variations in marker expression, consistent with standard flow cytometry practices. Representative immune and non-immune gating strategies, utilizing the spleen as a reference organ, are shown (Figs 1A and B). Following exclusion of debris, doublets, and dead cells, CD45^+^ immune cells and CD45 ^−^ non-immune cells were separated, allowing parallel analysis of lymphoid, myeloid, and stromal-associated compartments, respectively. Within the immune compartment, conventional αβ T cells were identified as TCRβ^+^ TCRgd^−^ cells and further resolved into CD4^+^ and CD8 ^+^ subsets, whereas gd T cells were defined as TCRβ ^−^ TCRgd^+^ cells. B cells were identified within the TCRβ ^−^ TCRgd^−^ population based on CD19 and CD45R expression, including both CD19 ^+^ CD45R^+^ double-positive and single-positive subsets. Within the remaining CD19 ^−^ CD45R^−^ fraction, CD138^+^ plasma cells and NK1.1 ^+^ NK cells were identified. Among CD11b^+^MHC II ^+^ NK1.1^−^ Ly6G^−^ cells, CD11c^+^ dendritic cells were detected, whereas within the CD11b^−^MHC II^⁻^ compartment, F4/80^+^ macrophages were identified. Within the CD11b^−^MHC II ^−^ FcεRI^−^Siglec-F^−^ fraction, CD127 ^+^ CD90 ^+^ cells corresponded to innate lymphoid cells, which were further subdivided into CD117 ^+^ CD44 ^+^ innate lymphoid cell type 2 (ILC2) counterparts. Notably, CD44 was utilized within this framework as a representative secondary activation and memory marker to capture distinct tissue-resident subsets. Within the myeloid compartment, Ly6G^+^Ly6C^−^ cells were classified as neutrophils and Ly6G^−^Ly6C^+^ cells as monocytes. Among Ly6^−^Ly6C^−^ cells, eosinophils were identified as FcεRI^−^Siglec-F^+^ cells, while FcεRI⁺Siglec-F^−^ cells were further separated into CD117 ^+^ mast cells and CD117 ^−^ basophils. Within the CD45 ^−^ compartment, epithelial cells were identified as CD326^+^ cells, endothelial cells as CD31^+^ cells, fibroblasts as CD140a^+^ cells, and neuronal-associated cells as CD56 ^+^ CD90 ^+^ cells. While CD90 serves as a shared marker across both immune and non-immune compartments, these distinct populations were effectively resolved through the mutually exclusive expression of cell-type-specific markers. This framework was further supported by UMAP visualization, in which cell populations defined by the established gating strategy formed distinct distributions in representative tissues such as bone marrow, peritoneal lavage fluid, and lamina propria (Fig 1C). Thus, the panel provided a unified framework for broad cellular profiling across multiple murine tissues.

**Fig 2:**
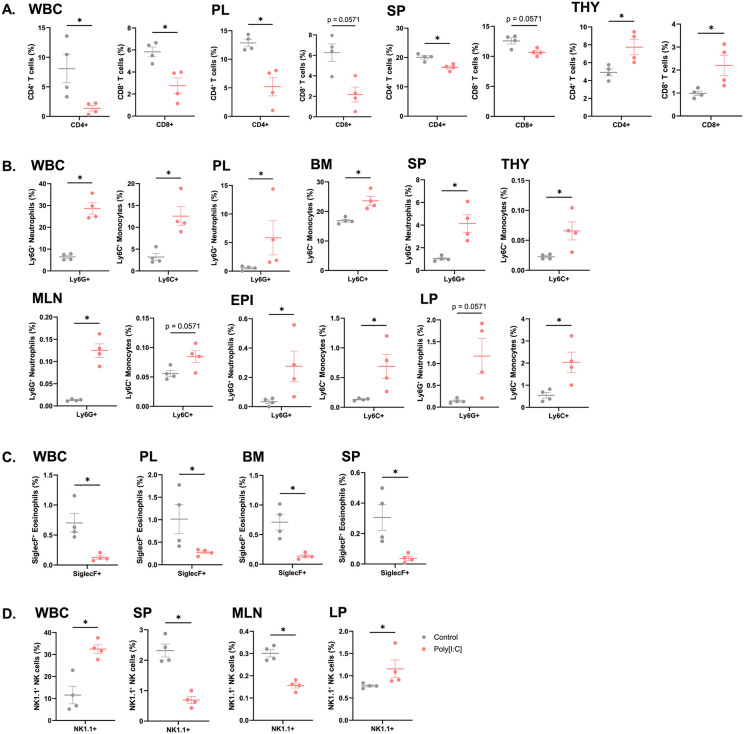
Cellular responses across tissues following poly(I:C) treatment. (A) Frequencies of CD4⁺ and CD8 ⁺ T cells across the indicated tissues following poly(I:C) treatment. Populations were analyzed within the CD45 ⁺ /CD4⁺ and CD45 ⁺ /CD8 ⁺ compartments. (B) Frequencies of Ly6G⁺ neutrophils and Ly6C⁺ monocytes across the indicated tissues following poly(I:C) treatment. Populations were analyzed within the CD45 ⁺ /Ly6G⁺ and CD45 ⁺ /Ly6C⁺ compartments. (C) Frequencies of Siglec-F⁺ eosinophils across the indicated tissues following poly(I:C) treatment. Populations were analyzed within the CD45 ⁺ /Siglec-F⁺ compartment. (D) Frequencies of NK1.1 ⁺ NK cells across the indicated tissues following poly(I:C) treatment. Populations were analyzed within the CD45 ⁺ /NK1.1 ⁺ compartment. WBC, white blood cells; PL, peritoneal lavage; BM, bone marrow; SP, spleen; THY, thymus; MLN, mesenteric lymph node; EPI, intestinal epithelium; LP, lamina propria. Each dot represents an individual mouse (*n = 4* mice per group). Bars indicate mean ± SEM. Statistical significance was assessed using the Mann–Whitney test. *p < 0.05.

To evaluate the biological applicability, we applied the panel to an acute in vivo poly(I:C) model. Poly(I:C), a synthetic double-stranded RNA analog widely used as a viral mimic, is a well-established inducer of antiviral innate immune activation, including type I interferon–driven responses, and broader systemic immune perturbation [31]. We therefore selected this model as a stringent test of whether our panel could capture coordinated immune remodeling across tissues in vivo. Using saline-treated and poly(I:C)-treated *Cyp11b1*^*mScarlet*^ reporter mice, we analyzed tissues 24 h after intraperitoneal injection. Broad immune cell changes were detected across multiple tissues, revealing substantial remodeling of systemic immune composition (Fig 2). In the peripheral blood, peritoneal lavage fluid, and spleen, poly(I:C) exposure was associated with reduced CD4+ and CD8+ T cell frequencies, consistent with early redistribution of peripheral lymphoid compartments during acute antiviral-like inflammation [32,33] (Fig 2A). In parallel, Ly6G+ neutrophils and Ly6C+ monocytes increased across multiple tissues, in line with previous reports showing early mobilization and accumulation of innate myeloid populations following poly(I:C) exposure [34–36] (Fig 2B). Siglec-F+ eosinophils also showed a broad downward trend across tissues, with significant reductions in peripheral blood, peritoneal lavage fluid, spleen, and bone marrow (Fig 2C). This pattern may reflect suppression or reprogramming of eosinophil-associated type 2 features under an antiviral/type 1-skewed inflammatory environment [37,38]. Together, these findings indicate that poly(I:C) drives a shift in immune composition away from a homeostatic lymphoid-dominant state toward a myeloid-biased inflammatory profile across tissues.

T cell responses were nevertheless compartment-specific. In contrast to the reduction in peripheral CD4+ and CD8+ T cells, thymic CD4+ and CD8+ T cell frequencies were maintained or increased, indicating that poly(I:C)-associated immune remodeling was not uniform across tissues (Fig 2A). Because the thymus is the primary site of T cell development, where single-positive thymocytes undergo further maturation before export [39,40], changes in thymic CD4+ and CD8+ frequencies may not directly reflect those observed in peripheral tissues. NK1.1+ NK cells also displayed tissue-dependent changes, with reduced frequencies in the spleen and the mesenteric lymph nodes but increased frequencies in the blood and the small-intestinal lamina propria, suggesting context-dependent redistribution or selective accumulation across compartments (Fig 2D). Poly(I:C) is known to activate NK cells in vivo [41], although the tissue distribution of this response may vary with inflammatory context. These observations further illustrate the advantage of this panel, which enables direct comparison of the same cellular populations across multiple tissues within a single experiment. Collectively, the results demonstrate that the panel can resolve both common and tissue-specific features of systemic and intestinal immune remodeling in a biologically relevant antiviral-like inflammatory setting.

**Fig 3:**
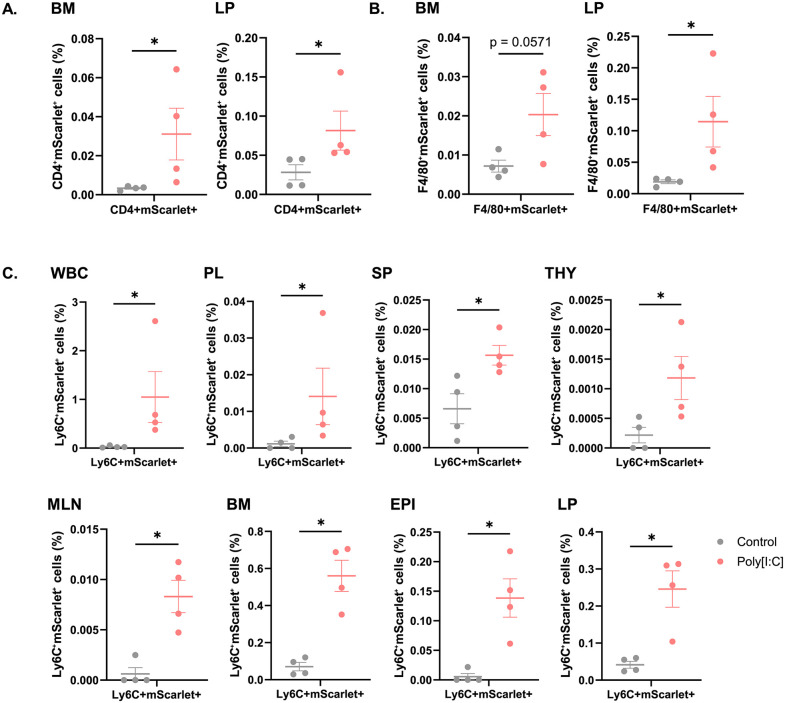
Compartment-specific mScarlet expression following poly(I:C) treatment. (A) Frequency of mScarlet⁺ cells in CD4 ⁺ immune cells in bone marrow and lamina propria following poly(I:C) treatment. Populations were analyzed within the CD45 ⁺ /CD4 ⁺ compartment. (B) Frequency of mScarlet⁺ cells in F4/80 ⁺ immune cells in bone marrow and lamina propria following poly(I:C) treatment. Populations were analyzed within the CD45 ⁺ /F4/80 ⁺ compartment. (C) Frequency of mScarlet⁺ cells in Ly6C⁺ immune cells across tissues following poly(I:C) treatment. Populations were analyzed within the CD45 ⁺ /Ly6C⁺ compartment. WBC, white blood cells; PL, peritoneal lavage; SP, spleen; THY, thymus; MLN, mesenteric lymph node; BM, bone marrow; EPI, intestinal epithelium; LP, lamina propria. Each dot represents an individual mouse (*n = 4* mice per group). Bars indicate mean ± SEM. Statistical significance was assessed using the Mann–Whitney test. *p < 0.05.

Beyond the broad immune changes detected across tissues, the panel also allowed endogenous mScarlet fluorescence to be analyzed in the same poly(I:C)-treated *Cyp11b1*^*mScarlet*^ reporter mice alongside antibody-based immune phenotyping, thereby enabling a full 27-color panel. Given that poly(I:C) elicits systemic inflammatory responses and is known to elevate circulating glucocorticoid levels [42,43], this model provides a relevant context to examine whether *Cyp11b1*-associated reporter activity could be detected beyond the adrenal gland. Consistent with this, we found that mScarlet⁺CD4⁺ and mScarlet⁺F4/80 ⁺ are significantly increased by poly(I:C) challenge in the bone marrow and lamina propria of small intestine (Figs 3A and B). These results indicate that T cells and macrophages may upregulate *Cyp11b1* expression during viral inflammation as previously described in tumors [44,45]. This increase in mScarlet⁺CD4 ⁺ T cells was therefore interpreted as a localized inflammatory response in bone marrow and lamina propria rather than a generalized T cell response. The F4/80 ⁺ pattern may reflect related changes along the monocyte–macrophage axis, in line with previous evidence that monocyte–macrophage lineage cells can participate in local glucocorticoid production [44]. By contrast, interestingly, we discovered that Ly6C⁺ monocytes exhibited a widespread increase in mScarlet expression across all tissues examined (Fig 3C and S10 Fig). While previous studies have primarily focused on changes in monocyte abundance and activation state, our data reveal a subset of monocytes characterized by mScarlet expression, suggesting the induction of *Cyp11b1* expression in these cells. These results point to Ly6C⁺ monocytes as a notable *Cyp11b1*-expressing immune population following poly(I:C) treatment. This observation provides a basis for further investigation into the functional role of these cells during systemic inflammation. Collectively, these findings demonstrate that the panel is sensitive enough to resolve subtle endogenous reporter-associated shifts alongside broad immune phenotyping across tissues in vivo.

## 4. Discussion

In this study, we developed and optimized a 27-color full-spectrum flow cytometry panel for broad immunophenotyping in mice using a 3-laser platform. While existing high-parameter panels, such as various OMIPs, have significantly advanced the field, they often necessitate 4- or 5-laser configurations and typically focus on either lymphoid or myeloid lineages within limited tissue types. In contrast, our panel achieves comparable high-dimensional depth using a standard 3-laser system, thereby enhancing practical accessibility for a broader range of research settings. A key strength of this panel is that it enables simultaneous analysis of diverse immune populations together with selected non-immune cell types within a single workflow. This design is particularly advantageous in settings where tissue remodeling involves coordinated changes across multiple cellular compartments rather than isolated shifts within a single lineage. Accordingly, the panel is configured to capture major lymphoid and myeloid populations while also incorporating the *Cyp11b1*^*mScarlet*^ reporter, thereby extending conventional phenotyping to include *Cyp11b1* expression profiles within local tissue environments. This integrated design distinguishes our approach from traditional benchmarking panels by providing a holistic view of systemic immune responses accompanied by tissue-specific remodeling, including inflammatory, infectious, and stress-associated conditions. More broadly it provides a practical approach for examining how immune and structural compartments are jointly reorganized across multiple tissues or anatomical layers within the same mouse.

Notably, because the fluorochrome layout is spectrally optimized, this panel can be readily adapted for alternative targets—including intracellular or cell-type-specific markers— simply by substituting antibodies without major spectral re-optimization. By preserving this established spectral backbone, one can seamlessly transition from broad immunophenotyping to deep mechanistic studies. For instance, functional readouts such as transcription factors or cytokines can be integrated without disrupting the overall spectral balance. Relying on this pre-optimized configuration minimizes fluorochrome spreading errors and batch effects, providing a highly standardized and reproducible framework ideal for longitudinal tracking or multi-center collaborative studies. Moreover, the successful addition of an mScarlet reporter to the original 26-parameter panel exemplifies the expandability of our panel design. This indicates that other fluorescent proteins or dyes can be appended to accommodate diverse experimental designs, while leveraging AI to minimize spectral overlap.

Beyond this flexibility, these high-parameter capabilities are achieved using only a 3-laser full-spectrum configuration, enhancing practical accessibility and obviating the reliance on more instrument-intensive platforms. Importantly, despite this hardware constraint, the generated high-dimensional data retains the necessary depth and resolution to fully power advanced analytical pipelines. Consequently, researchers can readily apply sophisticated dimensionality reduction and clustering algorithms—such as t-SNE (t-distributed Stochastic Neighbor Embedding), UMAP (Uniform Manifold Approximation and Projection), and trajectory inference—to dissect complex cellular networks using widely available cytometers.

In addition to its technical expandability and practical accessibility, the fundamental value of this high-dimensional panel lies in its capacity to capture complex biological and *in vivo* phenomena. We demonstrated its biological utility by uncovering the unexpected *Cyp11b1* expression in Ly6C⁺ monocytes during poly(I:C)-induced viral mimicry, highlighting these cells as a novel immune population potentially capable of *de novo* glucocorticoid synthesis. While traditionally recognized primarily as potent drivers of acute inflammation, the identification of these myeloid cells as potential regulators in tissue-protective networks highlights the plasticity and dual-functional nature of the innate immune system. This biological insight has profound implications for a wide range of systemic inflammatory syndromes, such as sepsis, severe respiratory viral infections, and therapeutic cytokine release syndrome. Furthermore, this *in vivo* approach is equally vital for studying localized pathologies that exert systemic immunological consequences. For instance, in solid tumors or severe mucosal infections, localized tissue distress often triggers profound systemic immune alterations. As diverse immune populations are recruited to the disease microenvironment, the overall immune landscape undergoes significant reorganization. Furthermore, by incorporating representative non-immune lineages—such as epithelial or endothelial cells—our panel captures the spatial context of these immune infiltrates within the structural tissue niche. By enabling the high-resolution tracking of these population-level landscapes across multiple organs, our profiling strategy provides a critical tool for identifying systemic immune signatures in both systemic and localized disease conditions.

Several limitations should also be noted. First, the panel is primarily designed for surface-marker–based profiling and does not include intracellular functional readouts such as cytokine production, transcription factor expression, or signaling activity. Thus, although it is effective for identifying changes in cellular composition, it is less suited for directly assessing functional states within each population. Second, this study utilized 6–8 week-old female mice to ensure experimental consistency and minimize confounding social stress factors, which can be more prevalent in co-housed male mice. However, we recognize the exclusion of male mice as a limitation, as immune responses and *Cyp11b1* expression dynamics can exhibit significant sex-specific differences. Future investigations including both sexes will be necessary to fully generalize these findings. Third, although it captures a broad range of populations, some cell types are represented at a relatively general level and may require further refinement depending on the specific biological question. Additional marker substitution or panel adjustment may therefore be necessary for finer subclassification of closely related subsets. Finally, although the panel was optimized to minimize spectral interference, residual spreading was not completely eliminated in all channels. This likely reflects, in part, the inherent challenge of generating ideal reference controls for every marker under practical experimental conditions. In the present study, single-color controls were derived from tissue or cell populations exhibiting the strongest apparent signal within the sampled materials, which supported robust unmixing overall but may still leave room for further refinement.

## 5. Conclusions

In summary, the panel presented here enables parallel high-dimensional analysis of multiple murine cell populations across distinct tissue compartments using a compact 3-laser full-spectrum configuration. Its successful application in an acute poly(I:C) inflammatory model showed that it can resolve coordinated immune remodeling across tissues while simultaneously capturing subtle reporter-associated cellular states. These features make it a practical platform for studying complex immune and tissue-associated heterogeneity across diverse experimental settings.

## Supporting information

S1 Fig Spectral characteristics of fluorochromes included in the panel. (A) Emission spectra of all fluorochromes. (B) Similarity matrix displaying pairwise spectral overlap values between fluorochromes. The Similarity Index ranges from 0 (no spectral overlap) to 1 (identical spectra); values ≤ 0.98 indicate acceptable distinction between channels. The Complexity Index quantifies the cumulative spectral interference across all fluorochromes, providing an overall measure of panel complexity and predicting potential signal spread and autofluorescence impact. (C) Spillover spreading matrix (SSM) showing fluorescence spillover between detection channels, used to optimize panel design and minimize spectral overlap. Spectral characteristics were generated using Cytek’s Full Spectrum Viewer (https://spectrum.cytekbio.com).
(PDF)

S2 Fig Spectral detection of mScarlet signal. (A) The emission spectrum of mScarlet is positioned between those of PE and PE-eFluor 610 (https://fluorofinder.com). (B) Gating strategy of spleen and adrenal gland cells from *Cyp11b1*^*mScarlet*^ reporter mice. Representative plots show sequential gating of Cells/Singlets/LIVE/mScarlet⁺ populations. In contrast to the spleen, mScarlet⁺ cells in the adrenal gland were detected mainly within the CD45 ⁻ fraction, consistent with reporter expression in non-immune parenchymal cells.
(PDF)

S3 Fig Evaluation of mScarlet signal specificity in the mouse adrenal gland. Flow cytometry plots showing mScarlet fluorescence intensity across different mouse genotypes. Full-panel-stained cells from wild-type mice (WT 1, 9 weeks old; WT 2, 6 weeks old) and *Cyp11b1*^*mScarlet*^ reporter mice (mSc 1 and mSc 2, 8 weeks old) were analyzed in parallel. The x-axis represents the animal genotype, and the y-axis represents mScarlet fluorescence intensity. Numbers above each plot indicate the percentage of parent events within the indicated gate.
(PDF)

S4 Fig Titration of antibodies. All used antibodies were titrated to determine optimal concentrations. The x-axes of each plot denote the concentration of antibodies used in each titration condition. The y-axes show the fluorescence intensity of the given fluorochrome.
(PDF)

S5 Fig Unmixing model. (A) Autofluorescence assessment after removing doublets and debris, showing the gate defining the high-AF population and its corresponding spectral intensity profile. (B) Plots displayed after gating on Cells/Singlets/Live/CD45^+^, illustrating two representative regions where standard Spectral Unmixing and Multiple AF Extraction differ in background removal.
(PDF)

S6 Fig FMO Controls. (A) Representative full-stain and APC (CD90) fluorescence-minus-one (FMO) control plots showing gating of CD19^+^CD45R^+^B cells within the TCRβ^−^TCRγδ^−^CD19^+^ compartment. Naïve B cells were identified from the CD19^+^CD45R⁺ population, whereas activated B cells and plasma cells were gated in parallel within the same parent gate. (B) Representative full-stain and fluorescence-minus-one (FMO) control plots for Spark NIR 685 (CD45R) and RB744 (CD117), showing identification of CD127^+^CD90^+^ cells within the TCRβ^−^TCRγδ^−^CD19^−^CD45R⁻NK1.1^−^FcεRI^−^ compartment. The right panels show the derived CD127^+^CD90^⁺^population.
(PDF)

S7 Fig Signal resolution in multicolor samples and single-reference controls. (A) Histograms of the single-reference controls (RC) are shown for each of the 26 markers. (B) Corresponding histograms from multicolor (MC)–stained splenocytes gated on Cells/Singlets/Live are shown using the same markers and scaling. (C) Overlay of RC (gray) and MC (pink) histograms provides a direct marker-by-marker comparison of signal concordance and mismatch. Larger deviations for selected markers, such as BV711 (CD326), reflect the use of a reference control derived from a different tissue source.
(PDF)

S8 Fig Unmixing Adjustment. Unmixing effects before and after adjustment are shown for events gated as Cells/Singlets/Live/CD45⁺ across multiple tissues. SP, spleen; THY, thymus; MLN, mesenteric lymph node; BM, bone marrow; PL, peritoneal lavage; WBC, white blood cells; EPI, intestinal epithelium; LP, lamina propria. Each pair of plots highlights a region where unmixing adjustmen**t** improves the signal distribution.
(PDF)

S9 Fig Comparison of marker MFI between multicolor samples and single-reference controls. Events gated on Cells/Singlets/Live/CD45 ⁺ are plotted on APC (CD90) and Alexa Fluor 647 (CD138) across multiple tissues. SP, spleen; THY, thymus; MLN, mesenteric lymph node; BM, bone marrow; PL, peritoneal lavage; WBC, white blood cells; EPI, intestinal epithelium; LP, lamina propria. A vertical cutoff (red line) derived from the reference control (RC) is applied to quantify the proportion of events exceeding this level in each multi-tissue control (MC), providing a visualization of pairwise compensation deviation for the APC–AF647 channel combination.
(PDF)

S10 Fig mScarlet expression in Ly6G⁺ cells following poly(I:C) treatment. Representative overlaid histograms showing mScarlet fluorescence intensity in CD45 ⁺ /Ly6G⁺ cells in BM and WBC from control and poly(I:C)-treated mice. Control, gray; poly(I:C), pink. BM, bone marrow; WBC, white blood cells.
(PDF)

S1 Table Instrument configuration and setup for the Cytek 3-laser Aurora. Laser and detector configurations followed the standard settings provided by the manufacturer. The table summarizes the center wavelength and bandwidth (nm) of each detector channel, along with representative fluorochromes commonly assigned to each detector.
(DOCX)

S2 Table Master mix for surface staining. Summary of dilution factors and volumes used to prepare the surface-staining master mix, with a total reaction volume of 50 µL.
(DOCX)

S3 Table Summary of markers and their classification. Summary of markers grouped into primary, secondary, and tertiary categories, with brief notes on their typical immunological roles.
(DOCX)
