# Supplementary material for: An optimized three-laser 27-color spectral flow cytometry panel for multi-organ profiling in mice
Source: PLoS One. 2026 Jul 20;21(7):e0347810. doi: 10.1371/journal.pone.0347810 (PMC13384274; doi:10.1371/journal.pone.0347810)
Supplement: S1 Fig — (A) Emission spectra of all fluorochromes. (B) Similarity matrix displaying pairwise spectral overlap values between fluorochromes. The Similarity Index ranges from 0 (no spectral overlap) to 1 (identical spectra); values ≤ 0.98 indicate acceptable distinction between channels. The Complexity Index quantifies the cumulative spectral interference across all fluorochromes, providing an overall measure of panel complexity and predicting potential signal spread and autofluorescence impact. (C) Spillover spreading matrix (SSM) showing fluorescence spillover between detection channels, used to optimize panel design and minimize spectral overlap. Spectral characteristics were generated using Cytek’s Full Spectrum Viewer (https://spectrum.cytekbio.com). (PDF) [file pone.0347810.s001.pdf]

A.

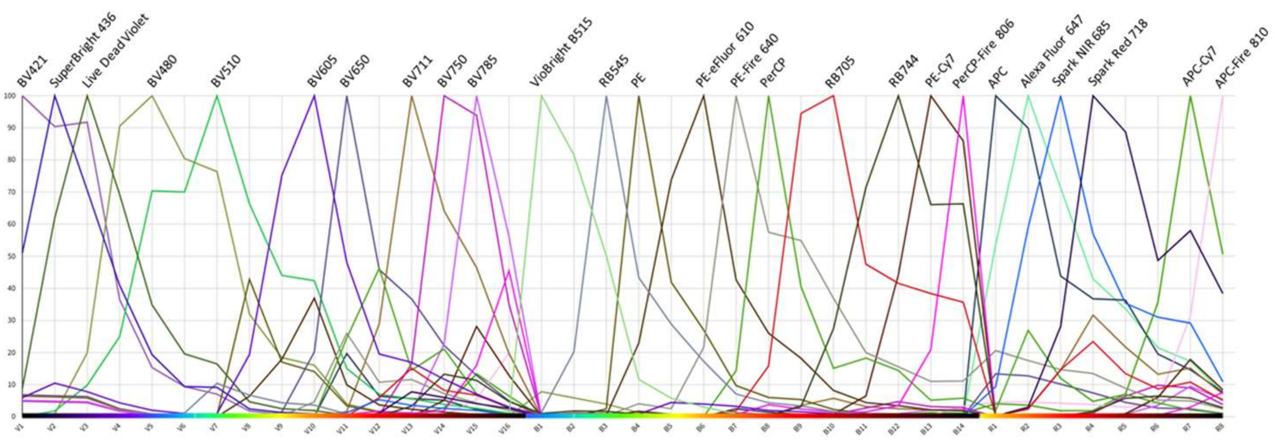

B.

	BV421	Super Bright 436	LIVE DEAD Violet	BV480	BV510	Super Bright 600	BV650	BV711	BV750	BV785	Vio Bright B515	RB545	PE	PE-eFluor 610	PE-Fire 640	PerCP	RB705	RB744	PE-Cy7	PerCP-Fire 806	APC	Alexa Fluor 647	Spark NIR 685	Spark Red 718	APC-Cy7	APC-Fire 810
BV421	1	0.95	0.79	0.27	0.16	0.11	0.1	0.08	0.06	0.08	0	0.01	0	0	0	0	0	0	0	0	0	0	0	0	0	0
Super Bright 436	0.95	1	0.87	0.34	0.21	0.12	0.09	0.08	0.06	0.08	0	0.01	0.01	0	0	0	0	0	0	0	0	0	0	0	0	0
LIVE DEAD Violet	0.79	0.87	1	0.57	0.36	0.12	0.08	0.06	0.05	0.06	0	0.02	0.02	0.01	0	0	0	0	0	0	0	0	0	0	0	0
BV480	0.27	0.34	0.57	1	0.86	0.19	0.06	0.03	0.02	0.02	0.08	0.09	0.1	0.05	0.01	0.01	0	0	0	0	0	0	0	0	0	0
BV510	0.16	0.21	0.36	0.86	1	0.44	0.16	0.06	0.04	0.03	0.01	0.1	0.21	0.12	0.03	0.04	0.01	0	0	0.01	0.02	0	0	0	0	0
Super Bright 600	0.11	0.12	0.12	0.19	0.44	1	0.52	0.19	0.12	0.08	0	0.08	0.24	0.34	0.15	0.16	0.04	0.02	0.02	0.03	0.06	0	0.01	0.01	0.01	0.01
BV650	0.1	0.09	0.08	0.06	0.16	0.52	1	0.46	0.25	0.16	0	0.01	0.05	0.12	0.27	0.4	0.09	0.04	0.04	0.05	0.32	0.19	0.18	0.12	0.06	0.05
BV711	0.08	0.08	0.06	0.03	0.06	0.19	0.46	1	0.68	0.46	0	0	0.01	0.03	0.19	0.3	0.23	0.15	0.15	0.15	0.21	0.19	0.29	0.38	0.21	0.15
BV750	0.06	0.06	0.05	0.02	0.04	0.12	0.25	0.68	1	0.82	0	0	0.01	0.01	0.08	0.18	0.11	0.17	0.22	0.23	0.08	0.03	0.08	0.13	0.19	0.13
BV785	0.08	0.08	0.06	0.02	0.03	0.08	0.16	0.46	0.82	1	0	0	0	0.01	0.05	0.09	0.08	0.12	0.25	0.34	0.04	0.02	0.05	0.09	0.22	0.2
Vio Bright B515	0	0	0	0.08	0.01	0	0	0	0	0	1	0.46	0.1	0.05	0.01	0	0.01	0.02	0	0	0	0	0	0	0	0
RB545	0.01	0.01	0.02	0.09	0.1	0.08	0.01	0	0	0	0.46	1	0.47	0.34	0.11	0.05	0.04	0.03	0.01	0.01	0	0	0	0	0	0
PE	0	0.01	0.02	0.1	0.21	0.24	0.05	0.01	0.01	0	0.1	0.47	1	0.57	0.17	0.08	0.05	0.02	0.02	0.01	0.01	0	0	0	0	0
PE-eFluor 610	0	0	0.01	0.05	0.12	0.34	0.12	0.03	0.01	0.01	0.05	0.34	0.57	1	0.48	0.25	0.15	0.06	0.04	0.03	0.02	0	0	0	0	0
PE-Fire 640	0	0	0	0.01	0.03	0.15	0.27	0.19	0.08	0.05	0.01	0.11	0.17	0.48	1	0.68	0.57	0.26	0.16	0.13	0.29	0.24	0.21	0.15	0.08	0.05
PerCP	0	0	0	0.01	0.04	0.16	0.4	0.3	0.18	0.09	0	0.05	0.08	0.25	0.68	1	0.46	0.22	0.12	0.11	0.24	0.23	0.22	0.11	0.05	0.04
RB705	0	0	0	0	0.01	0.04	0.09	0.23	0.11	0.08	0.01	0.04	0.05	0.15	0.57	0.46	1	0.61	0.41	0.29	0.12	0.13	0.18	0.21	0.11	0.08
RB744	0	0	0	0	0	0.02	0.04	0.15	0.17	0.12	0.02	0.03	0.02	0.06	0.26	0.22	0.61	1	0.78	0.5	0.03	0.02	0.04	0.08	0.08	0.05
PE-Cy7	0	0	0	0	0	0.02	0.04	0.15	0.22	0.25	0	0.01	0.02	0.04	0.16	0.12	0.41	0.78	1	0.73	0.03	0.03	0.05	0.09	0.18	0.15
PerCP-Fire 806	0	0	0	0	0.01	0.03	0.05	0.15	0.23	0.34	0	0.01	0.01	0.03	0.13	0.11	0.29	0.5	0.73	1	0.02	0.01	0.02	0.04	0.1	0.18
APC	0	0	0	0	0.02	0.06	0.32	0.21	0.08	0.04	0	0	0.01	0.02	0.29	0.24	0.12	0.03	0.03	0.02	1	0.92	0.7	0.43	0.2	0.16
Alexa Fluor 647	0	0	0	0	0	0	0.19	0.19	0.03	0.02	0	0	0	0	0.24	0.23	0.13	0.02	0.03	0.01	0.92	1	0.88	0.5	0.22	0.17
Spark NIR 685	0	0	0	0	0	0.01	0.18	0.29	0.08	0.05	0	0	0	0	0.21	0.22	0.18	0.04	0.05	0.02	0.7	0.88	1	0.68	0.32	0.21
Spark Red 718	0	0	0	0	0	0.01	0.12	0.38	0.13	0.09	0	0	0	0	0.15	0.11	0.21	0.08	0.09	0.04	0.43	0.5	0.68	1	0.54	0.39
APC-Cy7	0	0	0	0	0	0.01	0.06	0.21	0.19	0.22	0	0	0	0	0.08	0.05	0.11	0.08	0.18	0.1	0.2	0.22	0.32	0.54	1	0.68
APC-Fire 810	0	0	0	0	0	0.01	0.05	0.15	0.13	0.2	0	0	0	0	0.05	0.04	0.08	0.05	0.15	0.18	0.16	0.17	0.21	0.39	0.68	1

Complexity™ Index: 21.42

C.

	F4-80 | BV421	CD4 | Super Bright 436	Viability | LIVE DEAD Violet	CD56 | BV480	CD8 | BV510	CD140a | Super Bright 600	CD161 (NK1.1) | BV650	CD326 (Ep-CAM) | BV711	TCR gd | BV750	CD31 (PECAM-1) | BV785	FceR1 | Vio Bright B515	Ly-6C | RB545	CD170 (Siglec-F) | PE	TCR b | PE-eFluor 610	MHC Class II (I-A+I-E) | PE-Fire 640	Ly-6G | PerCP	CD19 | RB705	CD117 (c-kit) | RB744	CD44 | PE-Cy7	CD11b | PerCP-Fire 806	CD90 (THY1) | APC	CD138 | Alexa Fluor 647	CD45R (B220) | Spark NIR 685	CD11c | Spark Red 718	CD127 (IL-7Ra) | APC-Cy7	CD45 | APC-Fire 810
F4-80 | BV421		5.07	3.59	1.72	1.34	0.24	0.13	0	0.12	0	0.32	0.26	0	0.11	0.19	0.14	0	0.15	0.53	0.21	0.24	0	0	0	0	0
CD4 | Super Bright 436	3.15		3.08	1.65	1.16	0.37	0	0	0.1	0.24	0.13	0.27	0.13	0.11	0	0	0.06	0	0	0	0	0	0	0	0.11	0.06
Viability | LIVE DEAD Violet	3.02	7.38		3.99	3.02	0.72	0.42	0.23	0.13	0.09	0.5	0.33	0.5	0.56	0.07	0.11	0.04	0.11	0.1	0.09	0.17	0.41	0.2	0	0	0.06
CD56 | BV480	0.65	1.47	1.49		2.55	1.14	0.67	0.3	0.35	0.33	0.92	0.78	1.31	0.67	0.33	0.12	0	0	0	0.05	0.51	0.81	0.54	0.2	0	0
CD8 | BV510	0.52	1.07	1.03	1.8		1.62	1.09	0.71	0.77	0.66	0.33	0.32	1.29	0.75	0.32	0.01	0.09	0.22	0.64	0.24	0.41	0.55	0	0.28	0.2	0
CD140a | Super Bright 600	0.93	1.81	0.91	0.78	0.92		1.89	1.31	1.4	1.26	0.25	0.29	0.89	1.76	1.27	0.64	0.41	0.33	0.59	0.42	0.64	0.89	0.54	0.29	0.02	0
CD161 (NK1.1) | BV650	0.97	1.4	0.8	0.33	0.73	0.73		1.97	1.98	1.7	0.21	0.25	0	0.17	0.94	0.69	0.2	0.16	0.68	0.52	2.78	4.12	2.67	0.89	0.69	0.4
CD326 (Ep-CAM) | BV711	0.84	1.23	0.72	0.32	0.59	0	0.39		3.31	3.15	0.3	0.17	0	0	0.39	0.52	0.92	0.68	1.69	1.14	1.23	2.66	2.6	2.34	1.53	0.98
TCR gd | BV750	0.74	1.04	0.59	0.25	0.59	0	0	1.2		4.14	0.31	0.4	0	0.33	0	0	0.2	0.93	2.19	1.51	0.16	0.58	0.56	0.58	1.32	0.81
CD31 (PECAM-1) | BV785	0.86	1.27	0.72	0.44	0.66	0	0.18	0.44	2.04		0.31	0.32	0	0	0	0	0.15	0.45	2.42	1.61	0.33	0.36	0	0.23	1.37	0.84
FceR1 | Vio Bright B515	0	0.2	0	0.19	0.56	0.14	0	0	0	0		2.27	2.03	1.06	0.65	0.23	0.17	0.14	0	0.15	0	0	0	0	0.02	0.03
Ly-6C | RB545	0	0	0	0.81	1.9	0.47	0.44	0	0.14	0.11	0.82		3.48	2.17	1.8	0.72	0.61	0.48	0.69	0.41	0	0	0	0	0	0
CD170 (Siglec-F) | PE	0	0.22	0.62	1.72	2.39	1.19	0.74	0.23	0.21	0.23	0.25	0.52		2.1	1.61	0.63	0.53	0.37	0.48	0.23	0.51	0.61	0.26	0.05	0	0
TCR b | PE-eFluor 610	0	0	0	0.23	0.16	3.06	0.86	0.4	0.36	0.33	0	0.22	1.72		3.16	1.28	1.03	0.74	1.05	0.5	0.88	1.06	0.43	0.09	0.1	0.12
MHC Class II (I-A+I-E) | PE-Fire 640	0	0	0	0	0	0.28	2.23	0.92	0.93	0.93	0.02	0.15	0.8	1.59		2.17	1.95	1.56	2.46	1.32	2.96	4.16	2.45	0.8	0.78	0.45
Ly-6G | PerCP	0	0	0	0	2.16	0	1.46	0.99	1.65	1.56	1.12	1.03	0	0	2.36		1.7	1.52	2.44	0.78	2.57	4.91	2.82	0.83	0.71	0.25
CD19 | RB705	0.15	0	0	0	0.43	0	0.72	3.31	1.48	1.65	0.34	0.32	0.36	0.27	0.87	1.34		3.13	5.58	3.03	1.66	3.52	3.58	1.81	1.4	0.86
CD117 (c-kit) | RB744	0	0	0.13	0	0	0.31	0.38	0.69	2.97	2.21	0.16	0.31	0.49	0.33	0.33	0.49	1.35		7.58	4.32	0	0.43	0.56	0.69	1.29	0.84
CD44 | PE-Cy7	0.22	0.19	0	0	0	0	0	0.16	0.87	3.56	0.1	0.19	0.41	0.09	0.27	0.15	0.4	1.66		3.55	0	0	0.12	0.15	1.52	0.86
CD11b | PerCP-Fire 806	0.17	0	0	0.08	0.42	0	0	0.64	1.94	5.05	0.24	0	0	0.09	0.64	0.58	0.61	1.13	4.86		0	0.15	0.11	0.2	0.87	1.18
CD90 (THY1) | APC	0	0	0	0	0	0	2.41	0.77	0.95	0.86	0	0	0.16	0	1.08	0.47	0.31	0.22	0.36	0.12		10.26	5.85	1.79	1.56	0.93
CD138 | Alexa Fluor 647	0	0	0	0	0	0	1.29	0.3	0.36	0.26	0	0.2	0	0.07	0.43	0.23	0.13	0	0	0	6.23		6.8	1.98	1.72	1.03
CD45R (B220) | Spark NIR 685	0	0.33	0.23	0	0	0	1	0.6	0.68	0.68	0.21	0.3	0	0	0.3	0.23	0.21	0.36	0.65	0.35	4.35	8.56		2.17	2.12	1.27
CD11c | Spark Red 718	0	0	0	0	0	0	0.14	0.96	1.07	0.96	0	0	0	0	0	0.17	0.19	0.16	0.65	0.42	1.59	3.42	3.4		2.95	1.99
CD127 (IL-7Ra) | APC-Cy7	0.22	0	0	0	0.4	0	0.33	0.28	0.77	1.85	0.31	0.29	0.21	0.02	0	0	0	0.26	1.12	0.77	1.48	2.25	1.37	0.61		2.41
CD45 | APC-Fire 810	0	0	0	0	0.75	0	0.25	0.18	0.68	1.8	0.48	0.51	0.14	0.33	0.35	0	0	0.21	1.45	1.19	1.7	2.58	1.6	0.57	2.12	
