# Supplementary figures and images for: An optimized three-laser 27-color spectral flow cytometry panel for multi-organ profiling in mice
Source: PLoS One. 2026 Jul 20;21(7):e0347810. doi: 10.1371/journal.pone.0347810 (PMC13384274; doi:10.1371/journal.pone.0347810)

**A.**

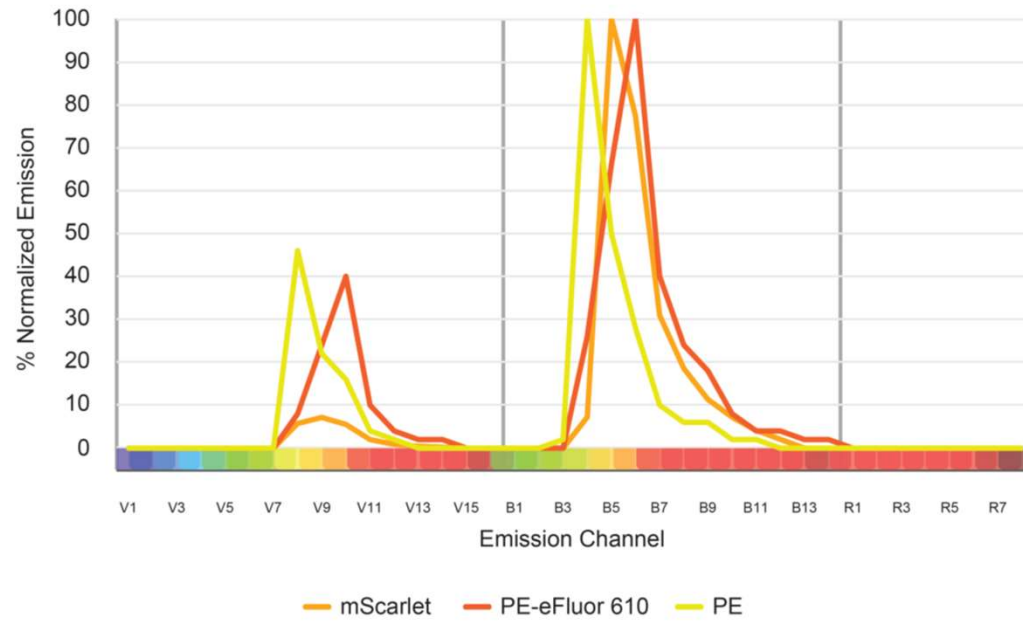

**B. Spleen**

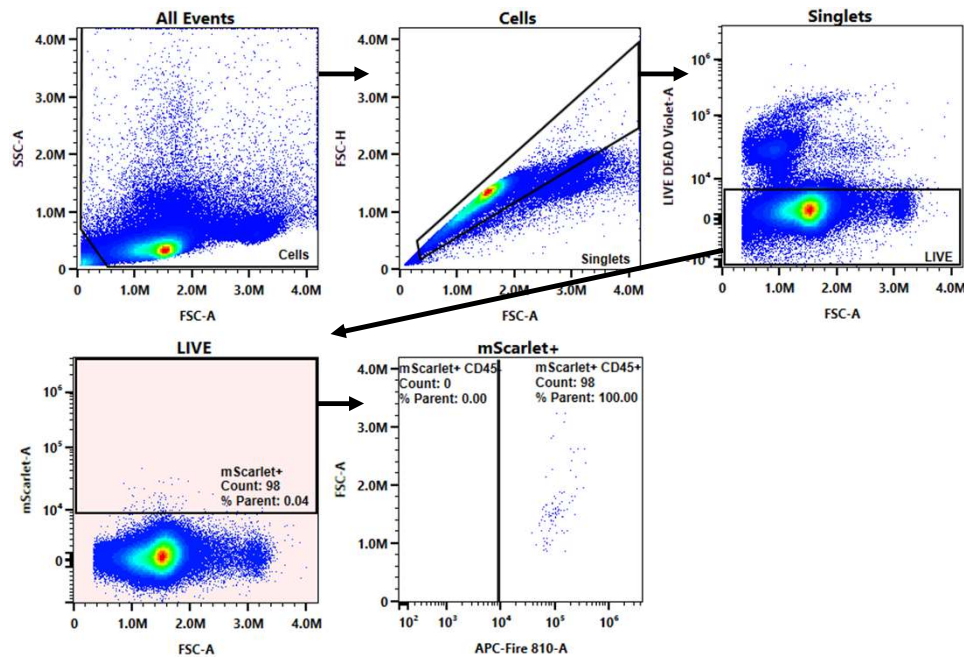

**Adrenal gland**

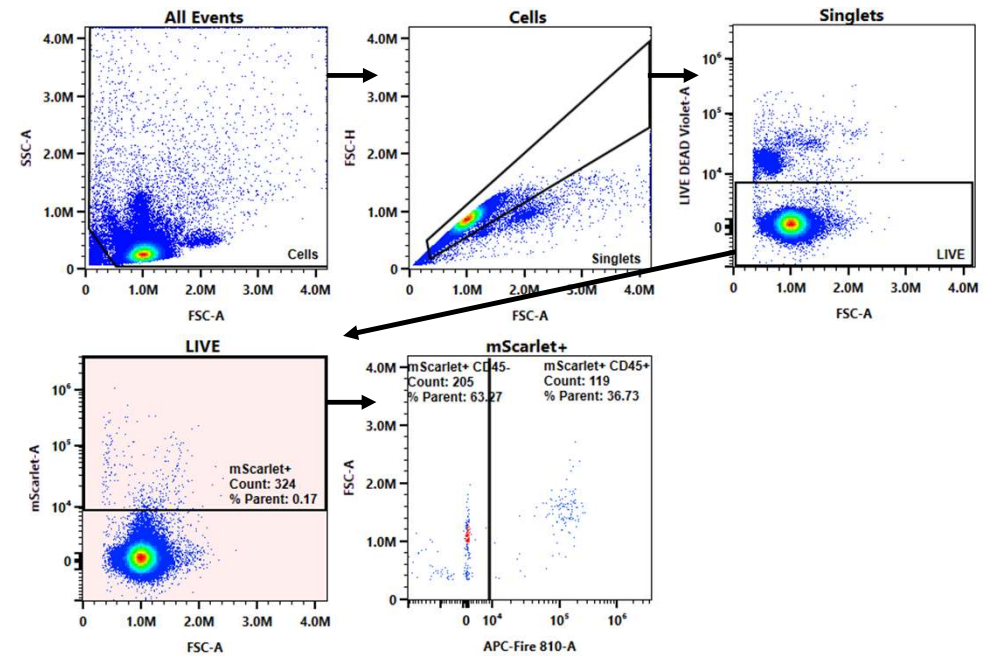

Supplement: S2 Fig — (A) The emission spectrum of mScarlet is positioned between those of PE and PE-eFluor 610 (https://fluorofinder.com). (B) Gating strategy of spleen and adrenal gland cells from Cyp11b1mScarlet reporter mice. Representative plots show sequential gating of Cells/Singlets/LIVE/mScarlet⁺ populations. In contrast to the spleen, mScarlet⁺ cells in the adrenal gland were detected mainly within the CD45 ⁻ fraction, consistent with reporter expression in non-immune parenchymal cells. (PDF) [file pone.0347810.s002.pdf]

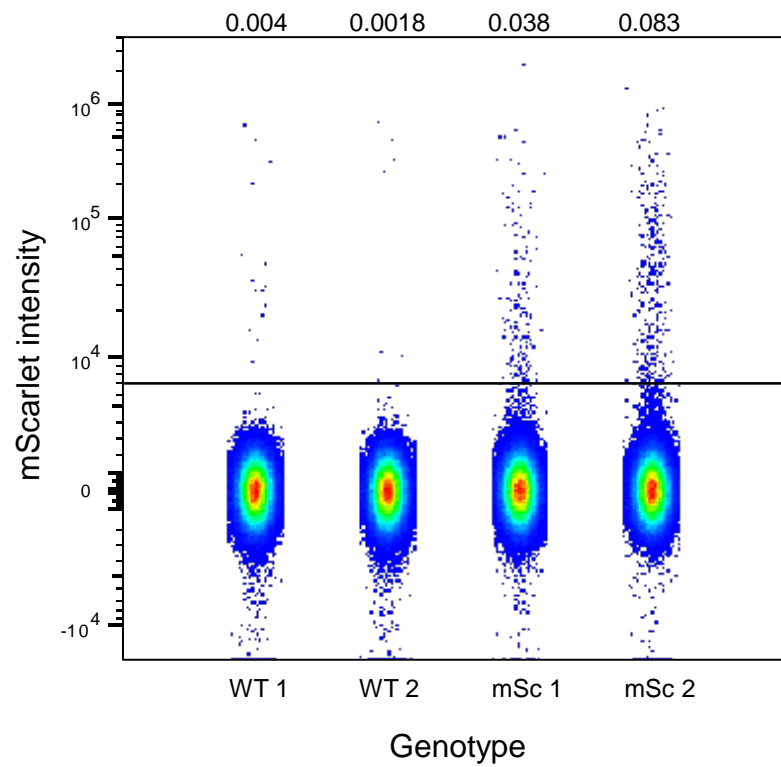

Supplement: S3 Fig — Flow cytometry plots showing mScarlet fluorescence intensity across different mouse genotypes. Full-panel-stained cells from wild-type mice (WT 1, 9 weeks old; WT 2, 6 weeks old) and Cyp11b1mScarlet reporter mice (mSc 1 and mSc 2, 8 weeks old) were analyzed in parallel. The x-axis represents the animal genotype, and the y-axis represents mScarlet fluorescence intensity. Numbers above each plot indicate the percentage of parent events within the indicated gate. (PDF) [file pone.0347810.s003.pdf]

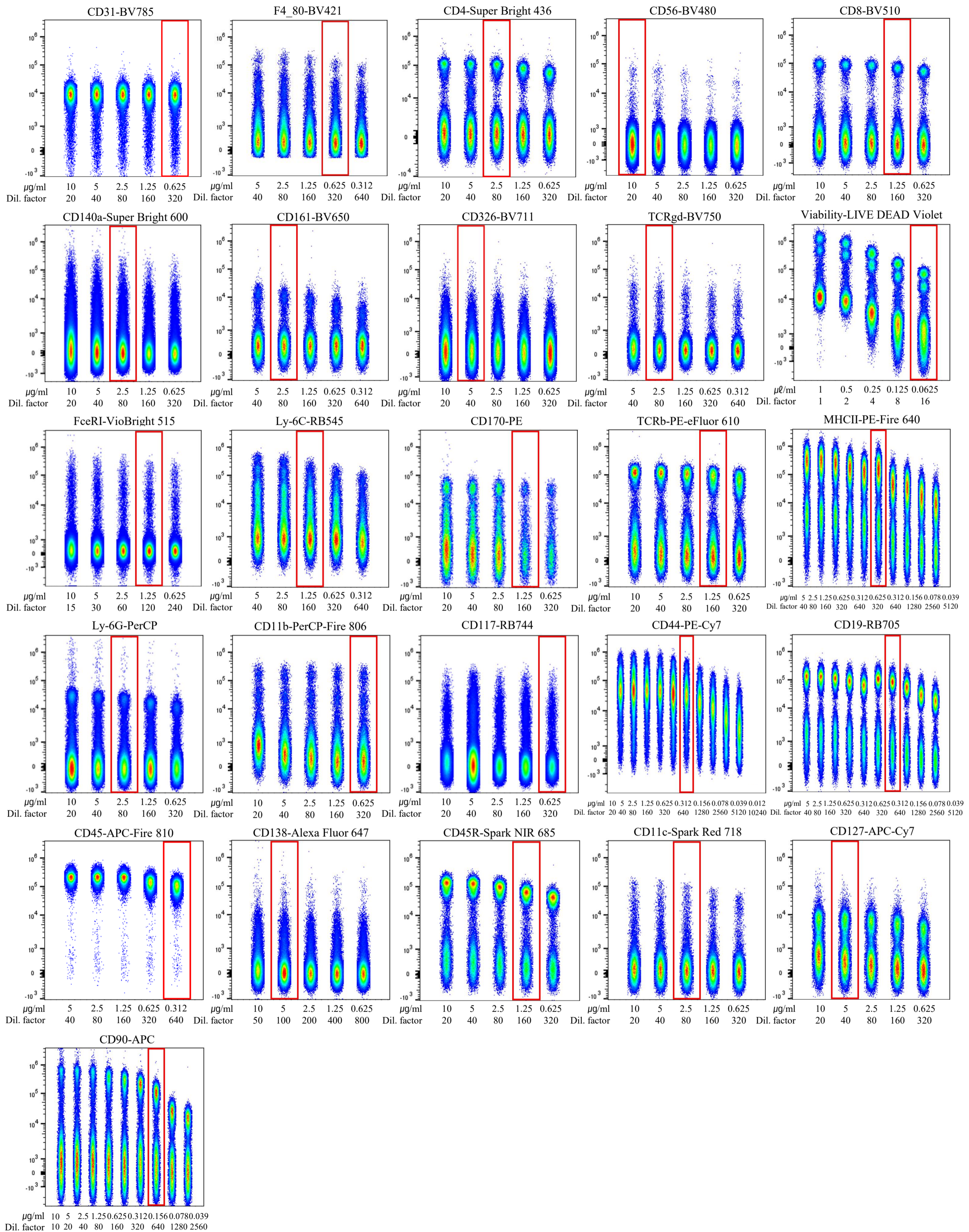

Supplement: S4 Fig — All used antibodies were titrated to determine optimal concentrations. The x-axes of each plot denote the concentration of antibodies used in each titration condition. The y-axes show the fluorescence intensity of the given fluorochrome. (PDF) [file pone.0347810.s004.pdf]

**A.**

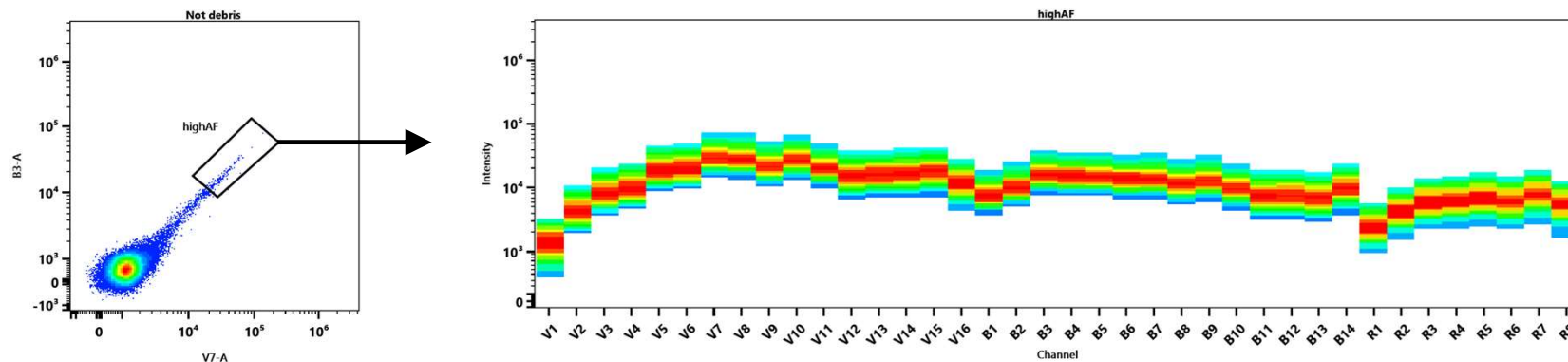

**B.**

## Spectral Unmixing Multiple AF Extraction

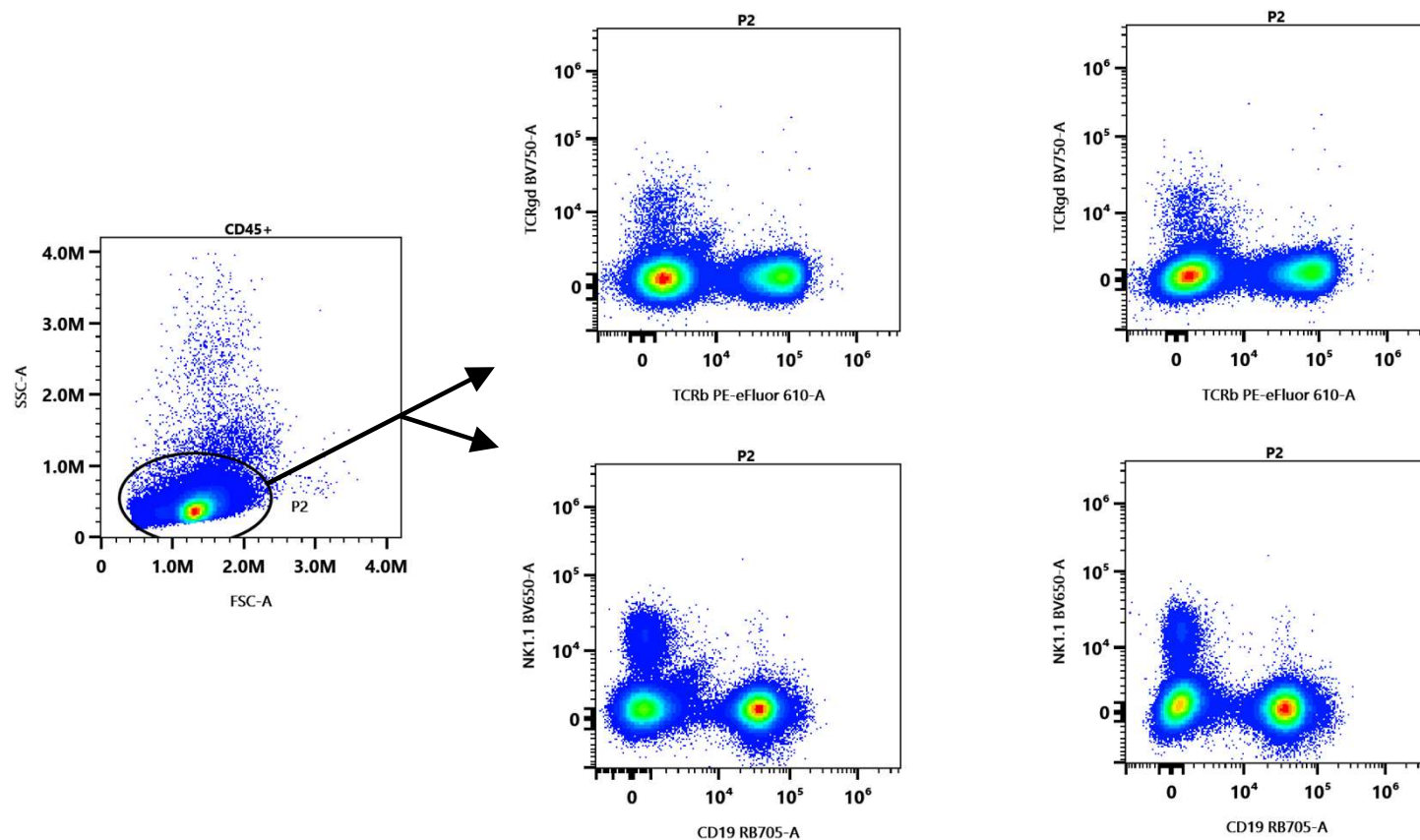

Supplement: S5 Fig — (A) Autofluorescence assessment after removing doublets and debris, showing the gate defining the high-AF population and its corresponding spectral intensity profile. (B) Plots displayed after gating on Cells/Singlets/Live/CD45+, illustrating two representative regions where standard Spectral Unmixing and Multiple AF Extraction differ in background removal. (PDF) [file pone.0347810.s005.pdf]

**A.**

**Full Stain**

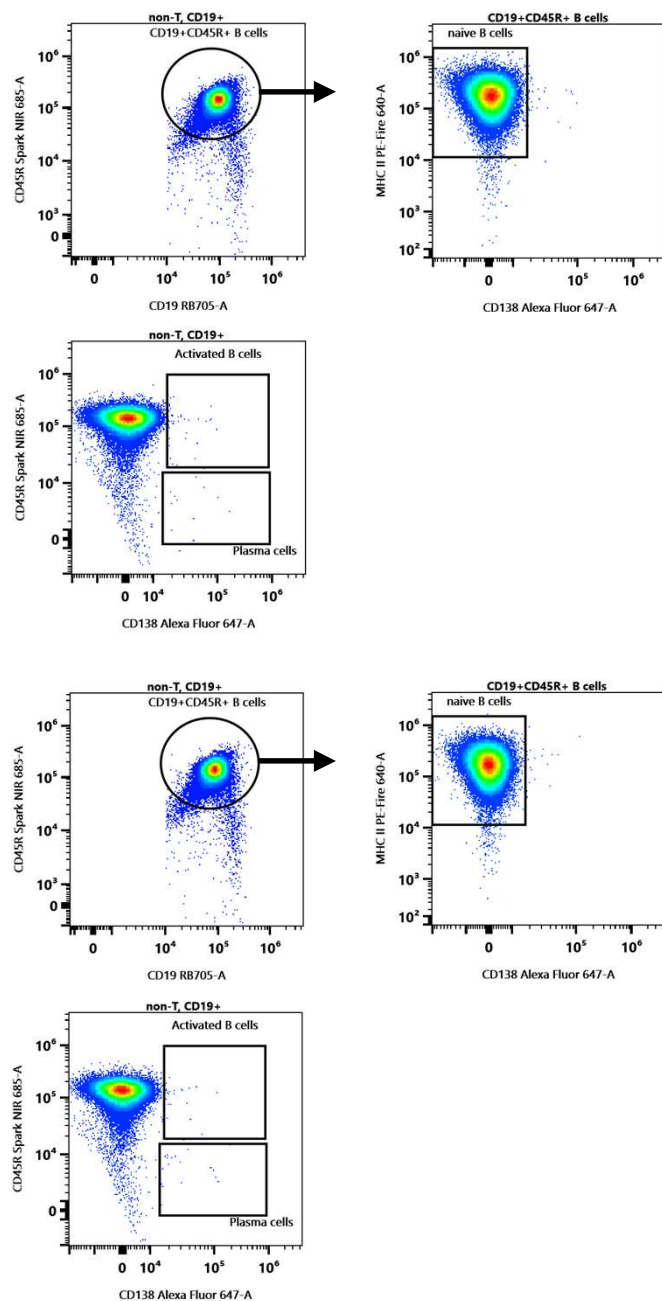

**FMO Control  
APC (CD90)**

**B.**

**Full Stain**

**FMO Control  
Spark NIR 685  
(CD45R)**

**FMO Control  
RB744 (CD117)**

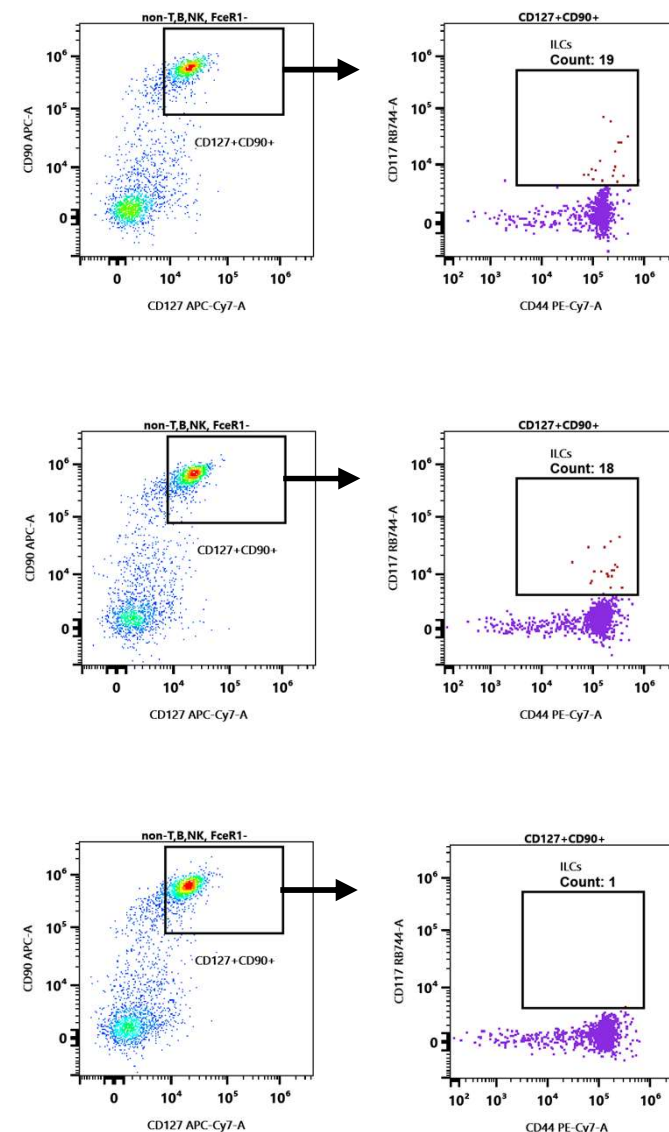

Supplement: S6 Fig — (A) Representative full-stain and APC (CD90) fluorescence-minus-one (FMO) control plots showing gating of CD19+CD45R+B cells within the TCRβ−TCRγδ−CD19+ compartment. Naïve B cells were identified from the CD19+CD45R⁺ population, whereas activated B cells and plasma cells were gated in parallel within the same parent gate. (B) Representative full-stain and fluorescence-minus-one (FMO) control plots for Spark NIR 685 (CD45R) and RB744 (CD117), showing identification of CD127+CD90+ cells within the TCRβ−TCRγδ−CD19−CD45R⁻NK1.1−FcεRI− compartment. The right panels show the derived CD127+CD90⁺population. (PDF) [file pone.0347810.s006.pdf]

A.

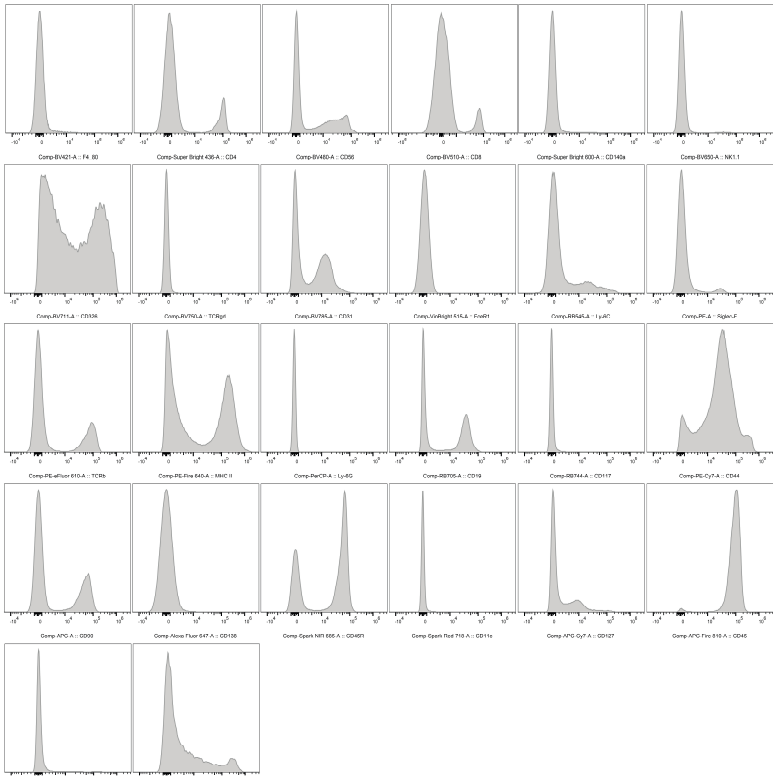

B.

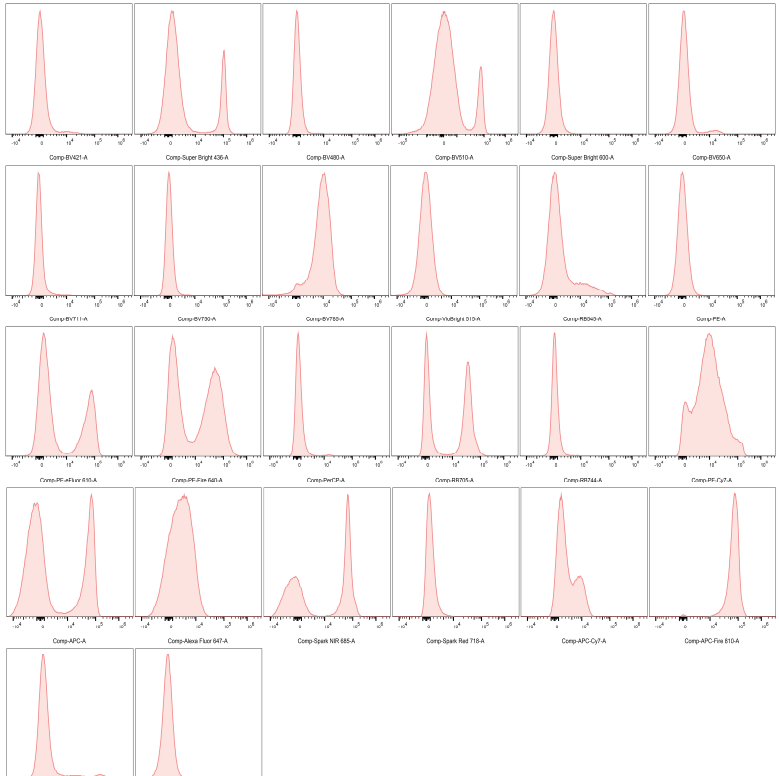

C.

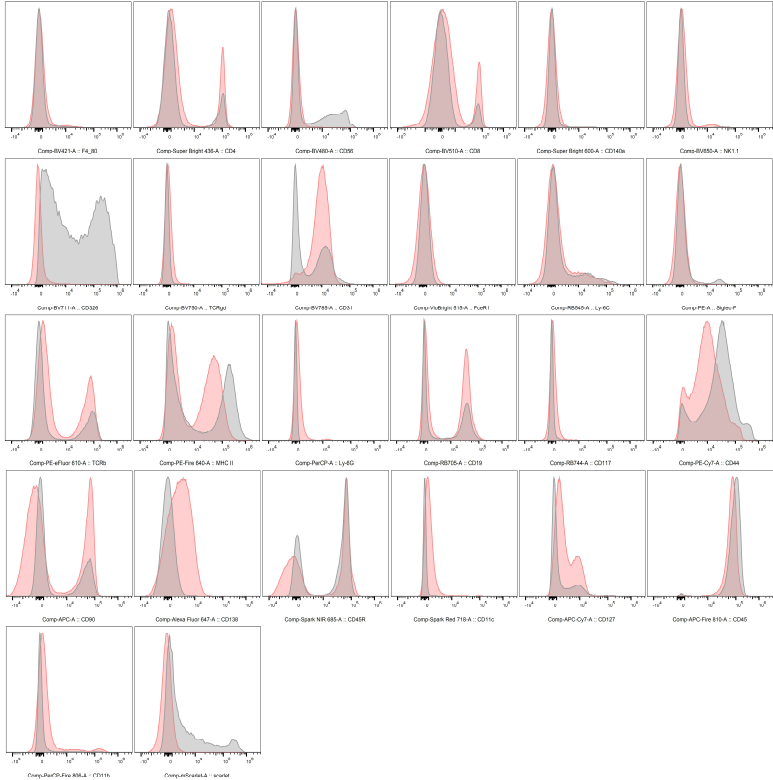

Supplement: S7 Fig — (A) Histograms of the single-reference controls (RC) are shown for each of the 26 markers. (B) Corresponding histograms from multicolor (MC)–stained splenocytes gated on Cells/Singlets/Live are shown using the same markers and scaling. (C) Overlay of RC (gray) and MC (pink) histograms provides a direct marker-by-marker comparison of signal concordance and mismatch. Larger deviations for selected markers, such as BV711 (CD326), reflect the use of a reference control derived from a different tissue source. (PDF) [file pone.0347810.s007.pdf]

Before  
correction

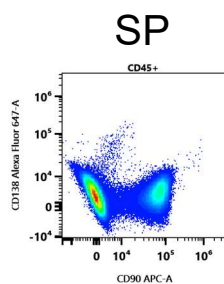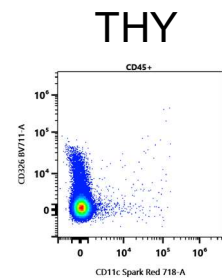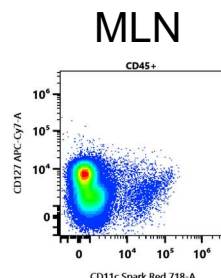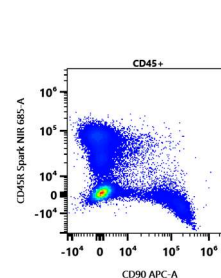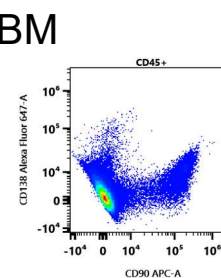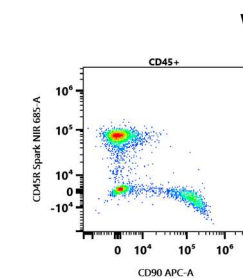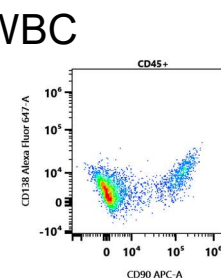

After  
correction

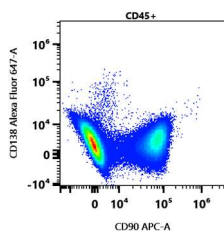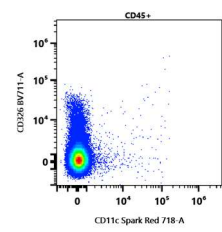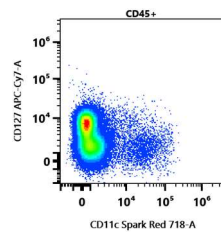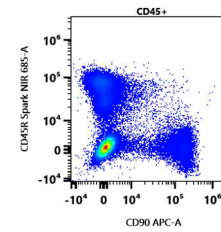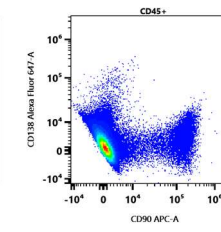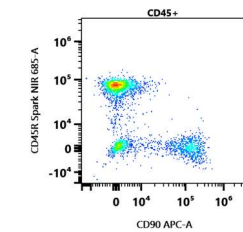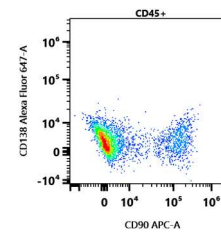

Before  
correction

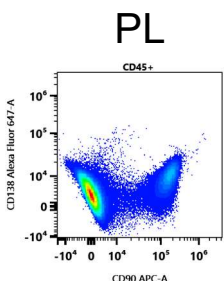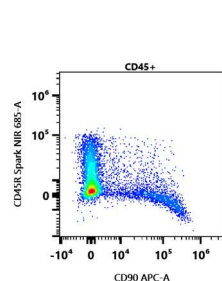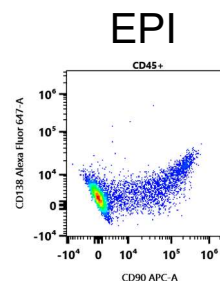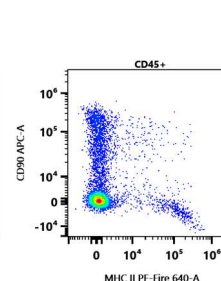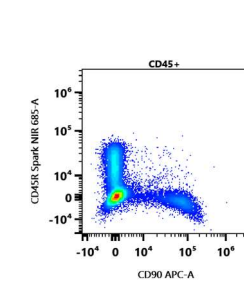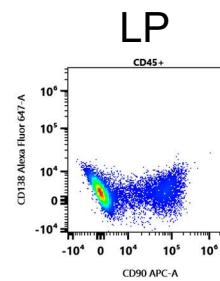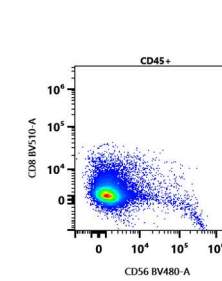

After  
correction

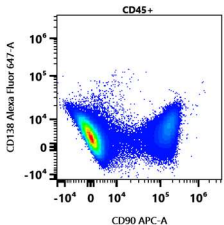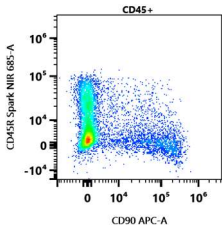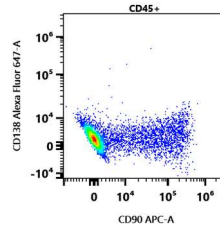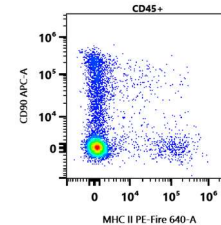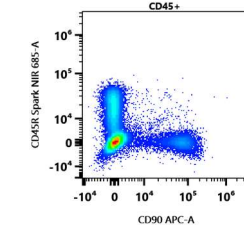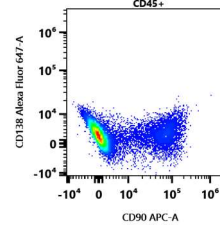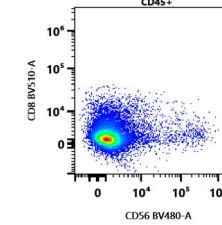

Supplement: S8 Fig — Unmixing effects before and after adjustment are shown for events gated as Cells/Singlets/Live/CD45⁺ across multiple tissues. SP, spleen; THY, thymus; MLN, mesenteric lymph node; BM, bone marrow; PL, peritoneal lavage; WBC, white blood cells; EPI, intestinal epithelium; LP, lamina propria. Each pair of plots highlights a region where unmixing adjustment improves the signal distribution. (PDF) [file pone.0347810.s008.pdf]

# Reference Control

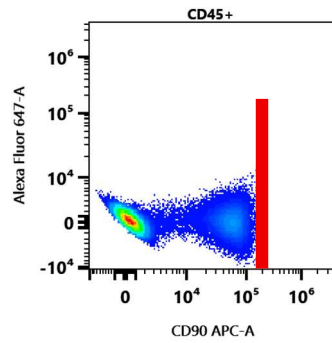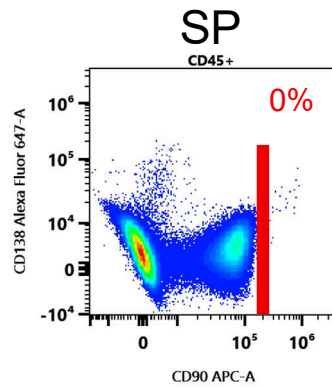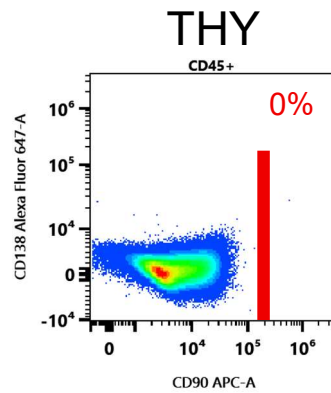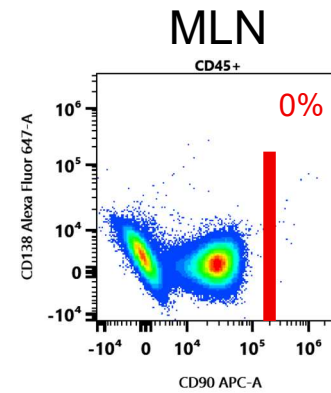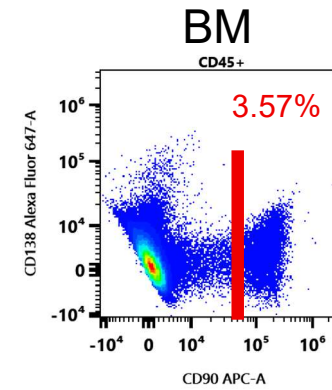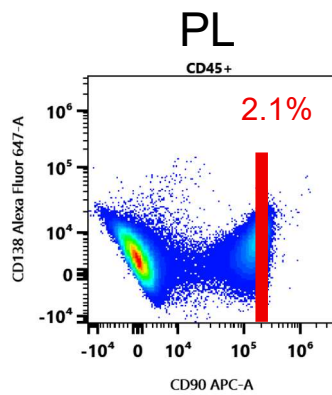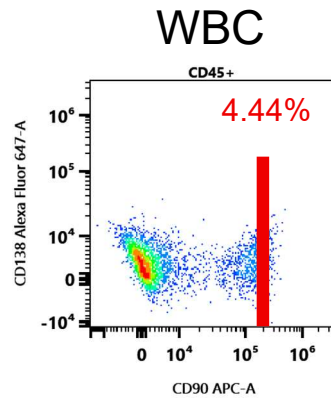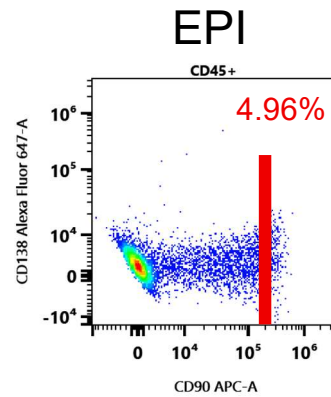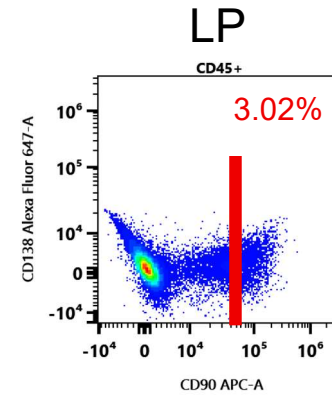

Supplement: S9 Fig — Events gated on Cells/Singlets/Live/CD45 ⁺ are plotted on APC (CD90) and Alexa Fluor 647 (CD138) across multiple tissues. SP, spleen; THY, thymus; MLN, mesenteric lymph node; BM, bone marrow; PL, peritoneal lavage; WBC, white blood cells; EPI, intestinal epithelium; LP, lamina propria. A vertical cutoff (red line) derived from the reference control (RC) is applied to quantify the proportion of events exceeding this level in each multi-tissue control (MC), providing a visualization of pairwise compensation deviation for the APC–AF647 channel combination. (PDF) [file pone.0347810.s009.pdf]

# BM

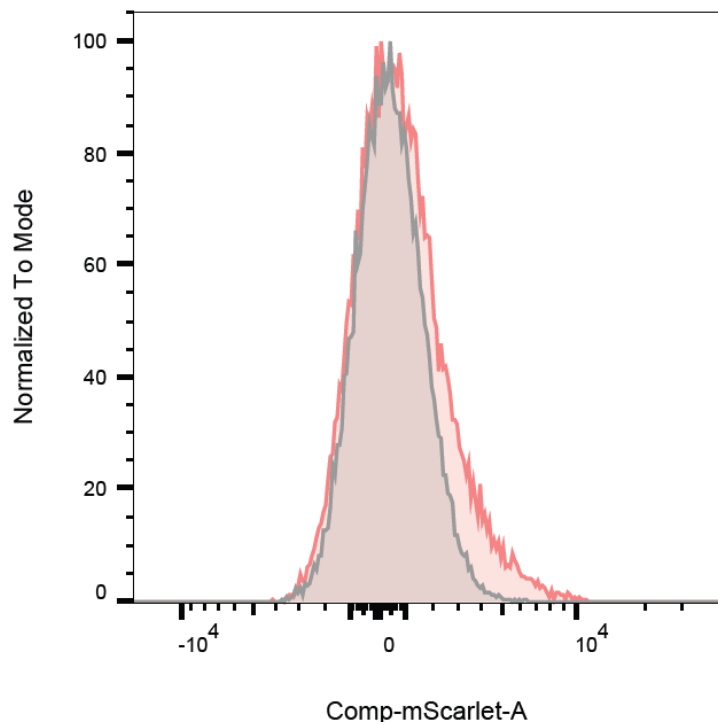

# WBC

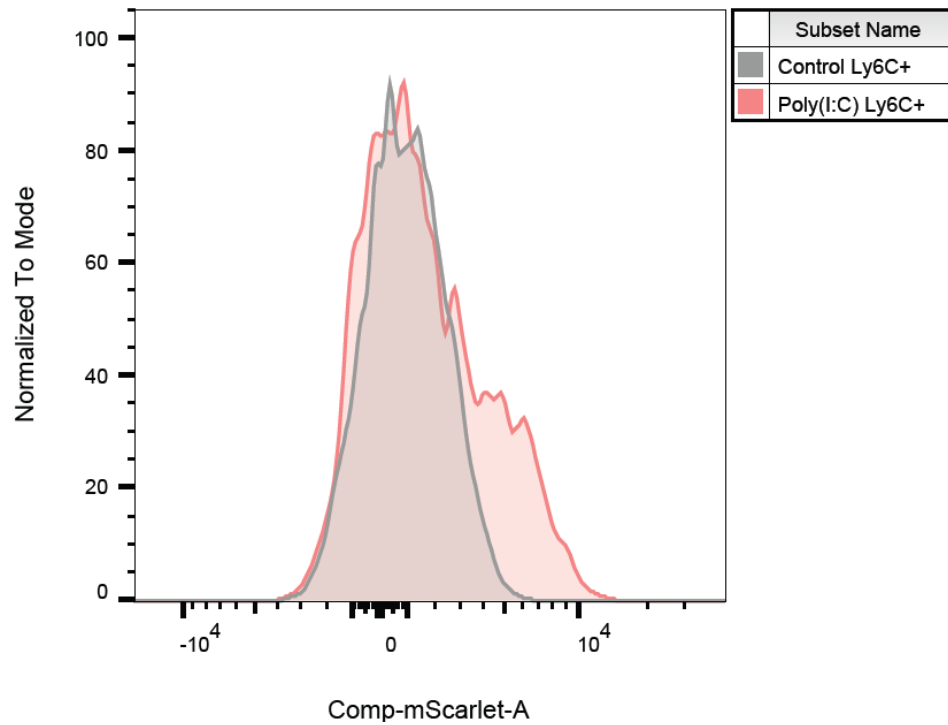

Supplement: S10 Fig — Representative overlaid histograms showing mScarlet fluorescence intensity in CD45 ⁺ /Ly6G⁺ cells in BM and WBC from control and poly(I:C)-treated mice. Control, gray; poly(I:C), pink. BM, bone marrow; WBC, white blood cells. (PDF) [file pone.0347810.s010.pdf]
