# Supplementary material for: An optimized three-laser 27-color spectral flow cytometry panel for multi-organ profiling in mice
Source: PLoS One. 2026 Jul 20;21(7):e0347810. doi: 10.1371/journal.pone.0347810 (PMC13384274; doi:10.1371/journal.pone.0347810)
Supplement: S1 Table — Laser and detector configurations followed the standard settings provided by the manufacturer. The table summarizes the center wavelength and bandwidth (nm) of each detector channel, along with representative fluorochromes commonly assigned to each detector. (DOCX) [file pone.0347810.s011.docx]

| **Violet (405nm: 100mW)** | | | |
| --- | --- | --- | --- |
|  |  |  |  |
| **Detector** | **Center**  **Wavelength (nm)** | **Bandwidth**  **(nm)** | **Fluorochrome** |
| V1 | 428 | 15 | BV421 |
| V2 | 443 | 15 | SuperBright 436 |
| V3 | 458 | 15 | Live Dead Violet |
| V4 | 473 | 15 |  |
| V5 | 508 | 20 | BV480 |
| V6 | 525 | 17 |  |
| V7 | 542 | 17 | BV510 |
| V8 | 581 | 19 |  |
| V9 | 598 | 20 |  |
| V10 | 615 | 20 | SuperBright 600 |
| V11 | 664 | 27 | BV650 |
| V12 | 692 | 28 |  |
| V13 | 720 | 29 | BV711 |
| V14 | 750 | 30 | BV750 |
| V15 | 780 | 30 | BV785 |
| V16 | 812 | 34 |  |
|  |  |  |  |
| **Blue (488nm: 50mW)** | | | |
|  |  |  |  |
| **Detector** | **Center**  **Wavelength (nm)** | **Bandwidth**  **(nm)** | **Fluorochrome** |
| B1 | 508 | 20 | VioBright B515 |
| B2 | 525 | 17 |  |
| B3 | 542 | 17 | RB545 |
| B4 | 581 | 19 | PE |
| B5 | 598 | 20 | mScarlet |
| B6 | 615 | 20 | PE-eFluor 610 |
| B7 | 661 | 17 | PE-Fire 640 |
| B8 | 679 | 18 | PerCP |
| B9 | 697 | 19 |  |
| B10 | 717 | 20 | RB705 |
| B11 | 738 | 21 |  |
| B12 | 760 | 23 | RB744 |
| B13 | 783 | 23 | PE-Cy7 |
| B14 | 812 | 24 | PerCP-Fire 806 |
|  |  |  |  |
| **Red (638nm: 80mW)** | | | |
|  |  |  |  |
| **Detector** | **Center**  **Wavelength (nm)** | **Bandwidth**  **(nm)** | **Fluorochrome** |
| R1 | 661 | 17 | APC |
| R2 | 679 | 18 | Alexa Fluor 647 |
| R3 | 697 | 19 | Spark NIR 685 |
| R4 | 717 | 20 | Spark Red 718 |
| R5 | 738 | 21 |  |
| R6 | 760 | 23 |  |
| R7 | 783 | 23 | APC-Cy7 |
| R8 | 812 | 34 | APC-Fire 810 |
