# Supplementary material for: An optimized three-laser 27-color spectral flow cytometry panel for multi-organ profiling in mice
Source: PLoS One. 2026 Jul 20;21(7):e0347810. doi: 10.1371/journal.pone.0347810 (PMC13384274; doi:10.1371/journal.pone.0347810)
Supplement: S2 Table — Summary of dilution factors and volumes used to prepare the surface-staining master mix, with a total reaction volume of 50 µL. (DOCX) [file pone.0347810.s012.docx]

| **Fluorochrome (marker)** | **Dilution factor** | **Volume [µL]**  **in master mix** |
| --- | --- | --- |
| APC (CD90, Thy-1) | 641 | 0.08 |
| APC-Cy7 (CD127, IL-7Rα) | 40 | 1.25 |
| APC-Fire 810 (CD45) | 641 | 0.08 |
| Alexa Fluor 647 (CD138) | 100 | 0.50 |
| Spark NIR 685 (CD45R, B220) | 160 | 0.31 |
| Spark Red 718 (CD11c) | 80 | 0.63 |
| BV421 (F4/80) | 320 | 0.16 |
| BV480 (CD56, NCAM) | 80 | 0.63 |
| BV510 (CD8) | 160 | 0.31 |
| BV650 (CD161, NK1.1) | 80 | 0.63 |
| BV711 (CD326, Ep-CAM) | 80 | 0.63 |
| BV750 (TCRγδ) | 80 | 0.63 |
| BV785 (CD31, PECAM-1) | 320 | 0.16 |
| Super Bright 436 (CD4) | 80 | 0.63 |
| Super Bright 600 (CD140a, PDGFRα) | 80 | 0.63 |
| PE (CD170, Siglec-F) | 160 | 0.31 |
| PE-Cy7 (CD44) | 641 | 0.08 |
| PE-eFluor 610 (TCRβ) | 160 | 0.31 |
| PE-Fire 640 (MHC II) | 320 | 0.16 |
| PerCP (Ly6G) | 80 | 0.63 |
| PerCP-Fire 806 (CD11b) | 320 | 0.16 |
| RB545 (Ly6C) | 160 | 0.31 |
| RB705 (CD19) | 641 | 0.08 |
| RB744 (CD117, c-Kit) | 320 | 0.16 |
| Vio Bright B515 (FcεRI) | 120 | 0.42 |
