# Supplementary material for: An optimized three-laser 27-color spectral flow cytometry panel for multi-organ profiling in mice
Source: PLoS One. 2026 Jul 20;21(7):e0347810. doi: 10.1371/journal.pone.0347810 (PMC13384274; doi:10.1371/journal.pone.0347810)
Supplement: S3 Table — Summary of markers grouped into primary, secondary, and tertiary categories, with brief notes on their typical immunological roles. (DOCX) [file pone.0347810.s013.docx]

| **Marker** | **Classification** | **Notes** |
| --- | --- | --- |
| CD45 | Primary | Strong leukocyte marker; distinct positive population. |
| TCRβ / TCRγδ | Primary | Lineage-defining T cell marker; clear on/off expression. |
| CD4 / CD8 | Primary | T cell subset markers; discrete populations. |
| CD19 / CD45R (B220) | Primary | B cell lineage markers with clear gating separation. |
| Ly6G | Primary | Neutrophil marker; bright and unambiguous. |
| Ly6C | Secondary | Monocyte subset marker; continuum from high to low expression. |
| CD44 | Secondary | Activation and memory marker with graded expression levels. |
| CD161 (NK1.1) | Secondary | Strain-dependent NK marker; expression intensity varies. |
| CD11b / CD11c / F4/80 | Secondary | Myeloid markers with overlapping expression patterns. |
| FcεRI | Secondary | Mast and basophil marker; expression varies by tissue and activation state. |
| MHC II | Secondary | Antigen-presenting cell activation marker; variable intensity. |
| CD31 (PECAM-1) | Secondary | Endothelial/hematopoietic marker; moderate continuous expression. |
| CD170 (Siglec-F) | Secondary | Eosinophil marker; bright in some tissues; may behave as Primary in eosinophil-rich organs. |
| CD56 (NCAM) | Tertiary | Neuronal cell marker; low to moderate expression. |
| CD90 (Thy-1) | Tertiary | T cell, ILC, and neuronal marker; low or rare expression expected. |
| CD140a (PDGFRα) | Tertiary | Fibroblast and mesenchymal marker; moderate to variable expression. |
| CD127 (IL-7Rα) | Tertiary | ILC marker; expressed on innate lymphoid cells and variably on memory T cells. |
| CD138 | Tertiary | Plasma cell marker; limited tissue expression and frequency. |
| CD117 (c-Kit) | Tertiary | Mast cell and basophil lineage marker; low frequency, dim signal. |
| CD326 (EpCAM) | Tertiary | Epithelial cell marker; low or variable expression in mixed tissues. |
