# Supplementary material for: An optimized three-laser 27-color spectral flow cytometry panel for multi-organ profiling in mice
Source: PLoS One. 2026 Jul 20;21(7):e0347810. doi: 10.1371/journal.pone.0347810 (PMC13384274; doi:10.1371/journal.pone.0347810)
Supplement: Attachment — Submitted filename: Response to Reviewers.pdf [file pone.0347810.s016.pdf]

Response to Reviewers

We would like to thank the reviewers for their positive and constructive comments on our manuscript.

Reviewer #1

(1) Scientific / Methodological Issues

(1.1) Question about the Statistical concerns

> *The study uses the Mann-Whitney U test throughout, which is appropriate for non-parametric comparisons. However, with only n= 4 to 5 mice per group (inferable from the dot plots in Figures 2 and 3), many comparisons yielding p=0.0571 are at the boundary of significance with this test minimum achievable p-value for these sample sizes. The authors should clarify exact n per group in the methods, which is currently absent. The lack of multiple-testing correction (e.g., Benjamini-Hochberg) is not addressed despite dozens of simultaneous comparisons across tissues and cell types, raising the risk of false positives.*
>
> *They use multiple tissue-by-cell-type comparisons and then report many p-values from Mann-Whitney tests, but I do not see any correction for multiple testing. That is a real weakness.*
>
> *Two specific problems that I foresee*
> *-many comparisons across tissues and populations inflate false-positive risk*
> *-the sample size appears small from the figures, yet the claims are broad*
>
> *At minimum I feel the authors should*
> *-state exact n per group in figure legends or methods*
> *-justify the statistical plan*
> *-address multiplicity, or explicitly say the analysis is exploratory*
>
> *Without that, some "significant" findings are less convincing.*

We agree with the reviewer's comments on the importance of statistical power and multiplicity. Accordingly, we have implemented the following clarifications:

- Sample Size: We have updated the Methods section (2.2) and Figure Legends (Figs. 2 and 3) to explicitly state the sample size.

- Exploratory Nature: As the primary objective of this study was the unbiased examination of immune subsets across 8 tissues, rather than within a given tissue, we approached the portion of data as an exploratory analysis. Consequently, to avoid Type II errors and the omission of potentially meaningful biological discoveries, p-values are reported without multiple-testing correction. However, for full transparency, the multiple-testing corrected results are also provided (please see below).
- Internal Validation via Cross-Tissue Consistency: To ensure findings were not false positives, we prioritized conserved phenotypic trends observed across multiple independent organs. While individual comparisons with n=4 may have limited power, the consistent directionality of these changes across 8 different tissues serves as a rigorous internal validation that minimizes the likelihood of stochastic noise.

Accordingly, we have explicitly stated the exploratory nature of this analysis and justified our statistical rationale in the Methods section (2.11) of the revised manuscript.

To further address the reviewer's concern, we also performed an additional multiple-comparison analysis using unpaired non-parametric Mann–Whitney U tests followed by the Benjamini, Krieger, and Yekutieli two-stage step-up false discovery rate (FDR, Q) procedure with Q set to 5%. Notably, this analysis was applied to the eight major immune cell populations within each tissue to assess potential cellular crosstalk. Even after FDR correction, spleen (SP) and White blood cells (WBC) showed discoveries across the eight major immune cell populations (Table R1). In contrast, the remaining six tissues did not meet the FDR discovery criterion (Table R2). We provide these results to demonstrate how the exploratory comparisons behave under a more conservative multiple-testing framework.

[Table R1. FDR-significant changes in major immune cell populations.]

Tissue	Cell type	Raw P value	FDR q value	Discovery
SP	$CD19^{+}CD45R^{+}$	0.028571	0.010000	Yes
	$CD4^{+}$	0.028571	0.010000	Yes
	$CD8^{+}$	0.057143	0.017143	Yes
	$CD127^{+}CD90^{+}$	0.028571	0.010000	Yes
	$NK1.1^{+}$	0.028571	0.010000	Yes
	Siglec-F^{+}	0.028571	0.010000	Yes
	$Ly6G^{+}$	0.028571	0.010000	Yes
	$Ly6C^{+}$	0.114286	0.030000	Yes
WBC	$CD19^{+}CD45R^{+}$	0.028571	0.004286	Yes
	$CD4^{+}$	0.028571	0.004286	Yes
	$CD8^{+}$	0.028571	0.004286	Yes
	$CD127^{+}CD90^{+}$	0.057143	0.007500	Yes

	NK1.1$^+$	0.028571	0.004286	Yes
	Siglec-F$^+$	0.028571	0.004286	Yes
	Ly6G$^+$	0.028571	0.004286	Yes
	Ly6C$^+$	0.028571	0.004286	Yes

[Table R2. Non-significant changes in major immune cell populations.]

Tissue	Cell type	Raw P value	FDR q value	Discovery
MLN	CD19$^+$CD45R$^+$	0.685714	0.720000	No
	CD4$^+$	0.057143	0.120000	No
	CD8$^+$	0.685714	0.720000	No
	CD127$^+$CD90$^+$	0.200000	0.280000	No
	NK1.1$^+$	0.028571	0.120000	No
	Siglec-F$^+$	0.114286	0.192000	No
	Ly6G$^+$	0.028571	0.120000	No
	Ly6C$^+$	0.057143	0.120000	No
BM	CD19$^+$CD45R$^+$	>0.999999	>0.999999	No
	CD4$^+$	0.057143	0.096000	No
	CD8$^+$	0.057143	0.096000	No
	CD127$^+$CD90$^+$	0.885714	>0.999999	No
	NK1.1$^+$	0.200000	0.280000	No
	Siglec-F$^+$	0.028571	0.096000	No
	Ly6G$^+$	0.057143	0.096000	No
	Ly6C$^+$	0.028571	0.096000	No
PL	CD19$^+$CD45R$^+$	0.114286	0.160000	No
	CD4$^+$	0.028571	0.080000	No
	CD8$^+$	0.057143	0.096000	No
	CD127$^+$CD90$^+$	0.685714	0.822857	No
	NK1.1$^+$	0.057143	0.096000	No
	Siglec-F$^+$	0.028571	0.080000	No
	Ly6G$^+$	0.028571	0.080000	No

Tissue	Cell type	Raw P value	FDR q value	Discovery
	Ly6C^{+}	0.885714	0.930000	No
LP	CD19^{+}CD45R^{+}	0.485714	0.680000	No
	CD4^{+}	>0.999999	>0.999999	No
	CD8^{+}	0.485714	0.680000	No
	CD127^{+}CD90^{+}	0.885714	>0.999999	No
	NK1.1^{+}	0.028571	0.120000	No
	Siglec-F^{+}	0.342857	0.680000	No
	Ly6G^{+}	0.057143	0.160000	No
	Ly6C^{+}	0.028571	0.120000	No
EPI	CD19^{+}CD45R^{+}	0.342857	0.582857	No
	CD4^{+}	0.685714	0.720000	No
	CD8^{+}	0.485714	0.582857	No
	CD127^{+}CD90^{+}	0.485714	0.582857	No
	NK1.1^{+}	0.114286	0.320000	No
	Siglec-F^{+}	0.485714	0.582857	No
	Ly6G^{+}	0.028571	0.120000	No
	Ly6C^{+}	0.028571	0.120000	No
THY	CD19^{+}CD45R^{+}	0.028571	0.060000	No
	CD4^{+}	0.028571	0.060000	No
	CD8^{+}	0.028571	0.060000	No
	CD127^{+}CD90^{+}	0.885714	0.930000	No
	NK1.1^{+}	0.200000	0.336000	No
	Siglec-F^{+}	0.342857	0.480000	No
	Ly6G^{+}	0.885714	0.930000	No
	Ly6C^{+}	0.028571	0.060000	No

(1.2) Question about the CD90 marker and gating strategy

The authors state that CD90 is used to mark T cells, ILCs, and neuronal cells simultaneously, but this creates an inherent ambiguity in gating, especially in the lamina propria where all

three populations coexist. The gating strategy relies on sequential marker exclusion to resolve this, but the manuscript does not explicitly address how CD90$^+$ CD56$^+$ neuronal cells are distinguished from CD90$^+$ CD127$^+$ ILCs when both reside in the CD45$^-$ and CD45$^+$ fractions respectively. This is a real interpretive concern that deserves direct acknowledgment.

The potential ambiguity arising from CD90 expression across multiple cell populations is an important consideration. While our gating framework utilizes CD45 to initially separate immune and non-immune fractions, the distinction between ILCs and neuronal-associated cells is further reinforced by their mutually exclusive expression of lineage-specific markers.

Specifically, ILCs are identified as CD45$^+$CD127$^+$CD56$^-$, whereas neuronal-associated cells are defined as CD45$^-$CD127$^-$CD56$^+$. Because these markers are analyzed in independent fluorescence channels, the risk of cross-contamination between the CD45$^+$ and CD45$^-$ fractions is effectively controlled.

We have added a clarifying sentence to the Results section to explicitly address this cross-exclusion logic.

(1.3) Question about the mScarlet reporter interpretation

The authors identify Ly6C$^+$ monocytes as "the most widespread and prominent glucocorticoid-producing immune population" based on elevated mScarlet expression. However, Cyp11b1 (11β-hydroxylase) encodes the enzyme for cortisol/corticosterone synthesis, but mScarlet reporter activity reflects Cyp11b1 promoter activation, not confirmed steroid secretion. The leap from reporter expression to "glucocorticoid production" is overstated without functional validation (e.g., intracellular steroid quantification, enzymatic activity assay). This should be softened to "Cyp11b1 reporter activity" throughout, or the distinction explicitly acknowledged.

It would be preferable to have orthogonal validation studies such as:
-steroid quantification from sorted populations
-CYP11B1 protein confirmation
-enzyme activity or pathway validation
-functional blockade or transcript-level confirmation in sorted cells

Two examples from the text:
-The abstract says they "identified a previously unrecognized, glucocorticoid-producing cell subset."
-The Results/Discussion state that Ly6C+ monocytes are "the most widespread and

prominent glucocorticoid-producing immune population."

We acknowledge the reviewer's critical point that mScarlet reporter activity, while reflecting *Cyp11b1* promoter activation, does not directly quantify downstream enzymatic activity or steroid secretion.

To ensure scientific accuracy and prevent any overstatement regarding functional glucocorticoid production, we have carefully revised the manuscript. Throughout the text, descriptions previously referring to these populations as "glucocorticoid-producing cells" or implying active steroid secretion have been changed to a more accurate, gene-level terms focusing strictly on *Cyp11b1* expression, upregulation, and reporter activity (e.g., "*Cyp11b1*-expressing cells" or "*Cyp11b1* expression profiles").

We believe these adjustments correctly align our terminology with the exact nature of the reporter system.

(1.4) Question about the Choice of Cell-based over Bead-based Controls

In section 2.6, the authors avoid compensation beads citing "subtle spectral discrepancies." While the cited references (16, 17) support this concern for some fluorochromes, the blanket avoidance of beads for all 26–27 fluorochromes is not standard practice. Cell-based controls can themselves introduce variability (especially autofluorescence) depending on cell health and tissue of origin. This trade-off is not discussed, and the choice warrants more nuanced justification.

The trade-off between bead- and cell-based controls is an important factor in high-parameter spectral flow cytometry. While compensation beads provide high signal intensity and zero background variance, cell-based controls were preferentially selected to utilize actual biological cells that reflect the internal environment of our experimental samples, rather than a plastic matrix. This approach effectively prevents subtle spectral discrepancies between synthetic beads and biological cells, which can cause cumulative unmixing errors in high-parameter panels.

To strictly control the inherent variability and baseline autofluorescence of cell controls, the vast majority of our single-stained references were standardized using splenocytes as a uniform, low-autofluorescence cellular background. For the remaining minor markers with low splenic expression, specific antigen-rich tissues were utilized solely to ensure positive signal accumulation. Furthermore, tissue-specific autofluorescence variations across the diverse target organs were comprehensively resolved via multiple AF extraction, as detailed in Methods section (2.9).

While our study successfully adopted cell-based controls, we fully agree that bead-based or hybrid strategies are equally valid. One can flexibly use either controls depending on their

specific marker expression levels and laboratory configurations.

We have updated the Methods section (2.6) to incorporate this justification.

(1.5) Question about the Rationale for a Uniform High-AF Extraction Model

> *The "Multiple AF Extraction (high AF)" model is applied uniformly across all tissues (Section 2.9), but the authors acknowledge that "subtle tissue-specific differences" exist. Applying a single high-AF model to tissues with variable autofluorescence (e.g., gut epithelium vs. peripheral blood) risks over-subtracting signal in low-AF samples. The rationale for this uniform approach over tissue-specific models is not adequately justified.*

We apologize for the confusion. The 'Multiple AF Extraction (high AF)' model was not a single standalone algorithm applied across different organs; rather, it was constructed independently for each specific tissue using its respective unstained control. Therefore, the model inherently accounts for the baseline autofluorescence variance of each distinct tissue type.

To ensure the validity of this approach, we confirmed that this tissue-specific application maintained adequate signal resolution without any significant loss of true positive signals. Even in low-AF tissues, such as peripheral blood, all expected cell populations were clearly observed, with no missing or unidentifiable populations.

We have updated the Methods section (2.9) to clarify this rationale.

(1.6) Question about the Interpretation of Cross-sectional Data as Dynamic

> *All biological data are collected at exactly 24 hours post-injection. The authors describe this as capturing "dynamic shifts," but a single time point does not constitute a dynamic analysis. The word "dynamic" is used repeatedly (abstract, results, discussion) yet is not supported by the experimental design, which is inherently cross-sectional.*

We agree that the term "dynamic" may overstate the temporal scope of our findings.

To address this feedback, we have revised the manuscript to replace the term "dynamic" with more precise descriptors such as "acute," "systemic," or "widespread," depending on the context. This adjustment ensures that our descriptions of immune responses and glucocorticoid production accurately reflect the specific timepoint, thereby avoiding any unintended implication of a multi-time-point kinetic analysis.

(1.7) Question about the Validity of mScarlet Detection without WT Controls

> *The mScarlet signal in $Ly6C^+$ monocytes is interpreted as biologically meaningful across all 8 tissues. However, Figure S9 (the only supplemental figure addressing $Ly6C^+$ mScarlet expression in non-target tissues) shows only bone marrow and peripheral blood histograms,*

with very modest histogram shifts. The lack of a wild-type (non-reporter) mouse negative control stained with the full panel in every tissue is a notable gap. mScarlet gating relies entirely on the Cyp11b1-mScarlet reporter mice, and spectral unmixing artifacts from PE/PE-eFluor 610 (which flank mScarlet's emission; Fig. S2A) in non-reporter cells cannot be formally excluded without a true negative control.

We thank the reviewer for the critical comment. We agree that establishing a true negative control is essential for validating these signals. Below, we address each point and present our adrenal gland control data to clarify the accuracy of our mScarlet gating:

1. Panel Validation using Adrenal WT Controls: Outside the adrenal gland, baseline mScarlet signals in the reporter mice are negligible (Taves, Matthew D. et al. *Science immunology*, 2023). To formally validate our 27-color panel, we have provided an additional parallel analysis using full-panel-stained WT controls and reporter mice in the adrenal gland (Fig R1).

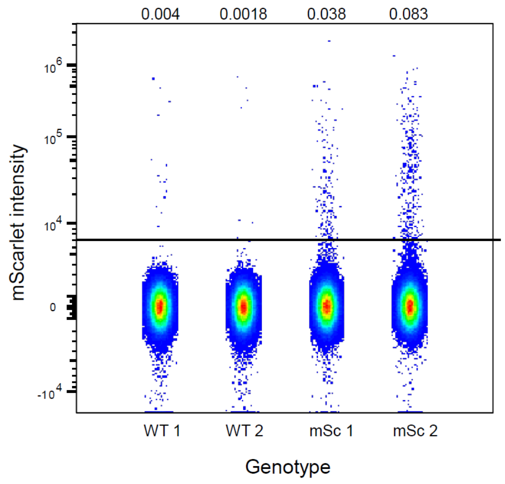

[Fig R1 (new S3 Fig). Evaluation of mScarlet signal specificity in the mouse adrenal gland.]

This newly provided adrenal data consists of LIVE/CD45$^-$ single cells. Crucially, the signals from the full-panel-stained non-reporter WT controls (WT 1 and WT 2) are largely confined below the baseline threshold of approximately 10^4, with no false-positive clusters splitting upward. Any spectral crossover or unmixing errors from the flanking PE or PE-eFluor 610 channels would have caused identical upward artifacts in these WT controls. This clear separation between the true reporter signal and the WT baseline supports the accuracy of our spectral unmixing matrix and mScarlet gating strategy.

2. Rationale for Figure S9 Presentation: Following *in vivo* poly(I:C) stimulation, we rigorously screened all 8 organs. Although the statistical analysis confirmed a significant increase in mScarlet expression within Ly6C$^+$ monocytes across all screened tissues, the visual shifts on histograms were very modest in most organs. To avoid potential confusion caused by presenting visually subtle plots, we selectively presented the bone marrow and peripheral blood

in Figure S9, as they displayed the most visually discernable shifts.

(1.8) Question about the Cross-Tissue Validation of Non-immune Gating

> *The non-immune gating strategy (Fig. 1B) is demonstrated only in the lamina propria. The paper claims the panel detects fibroblasts (CD140a^{+}), endothelial cells (CD31^{+}), epithelial cells (CD326^{+}), and neuronal cells (CD56^{+} CD90^{+}) across tissues, but validation in non-gut tissues for these populations is not shown. CD31 and, for instance, are also expressed on immune/hematopoietic cells (NK cells express CD56; hematopoietic progenitors express CD31), and the reliance on a CD45^{-} gate alone may be insufficient to exclude all contaminating immune populations.*

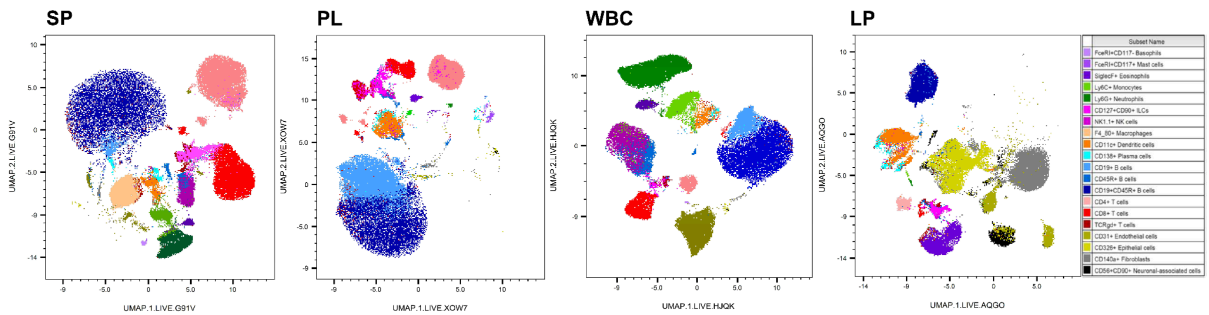

[Fig R2. Comparison of tissue-specific cell distributions.]

The lamina propria (LP) was intentionally selected as the representative tissue for Figure 1B because it is a tissue where all four major structural and stromal subsets we chose—fibroblasts, endothelial cells, epithelial cells, and neuronal cells—coexist in sufficient abundance to simultaneously demonstrate the full identification capacity of our panel. As illustrated in the tissue comparison (Fig R2), the proportion of non-immune cells is prominently and substantially higher in the LP compared to other sampled sites, such as the peritoneal lavage (PL), which consists almost entirely of hematopoietic immune populations.

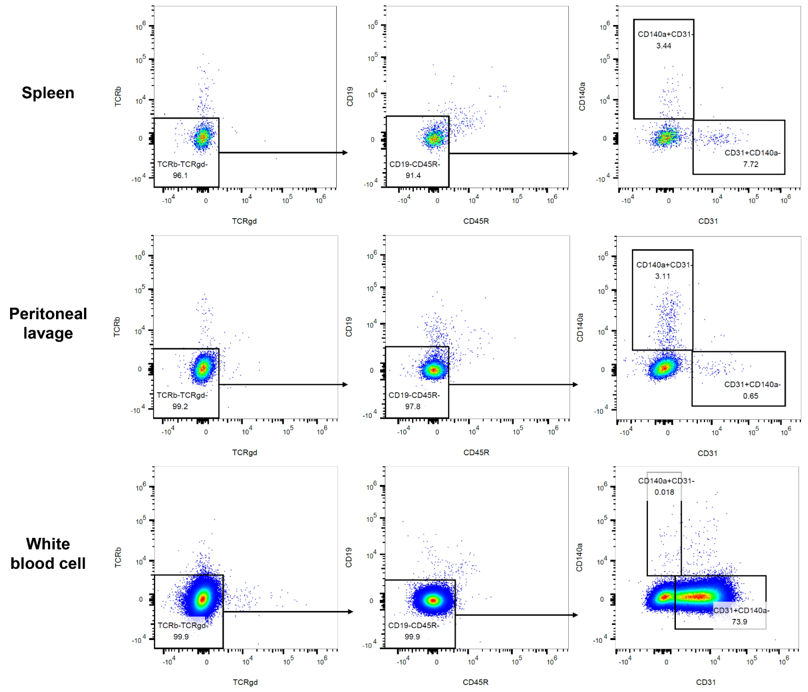

[Fig R3. Validation of the non-immune gating framework in diverse non-gut tissues.]

We acknowledge the limitations of identifying non-immune cells with just CD45 and a limited number of markers; however, our current gating strategy is still capable of distinguishing these non-immune cells to a reasonable extent. In this validation gating pipeline (Fig R3), we started from the CD45$^-$ population and sequentially isolated the double-negative fractions for T cells (TCRβ$^-$TCRγδ$^-$) and B cells (CD19$^-$CD45R$^-$). While this lineage-negative fraction initially represents an unspecified population of parenchymal cells, the subsequent populations can be evaluated with high confidence because potential immune cell contamination was rigorously eliminated through these upstream lineage markers. Within this framework, fibroblasts (CD140a$^+$) and endothelial cells (CD31$^+$) exhibit robust signal intensities and are resolved as distinct populations from the baseline double-negative parenchymal population. This data demonstrates that our CD45$^-$ gating strategy is technically sufficient to isolate pure non-immune cells without the risk of significant cellular contamination.

(1.9) Question about the Functional Rationale and Necessity of CD44

> *CD44 (PE-Cy7) appears in Table 1 and S2 as part of the panel and is used only to gate ILCs (CD117$^+$ CD44$^+$), yet it is classified as a "secondary" activation marker (Table S3). Its contribution to the major gating framework shown in Figure 1 is marginal, and the rationale for its inclusion over other potentially more discriminating markers is not discussed.*

We appreciate the reviewer's critical comment. CD44 was originally incorporated into our 27-color panel as a versatile secondary marker intended to examine the global activation and memory status rather than a lineage marker. Because CD44 typically exhibits a continuous and graded expression profile not a discrete on/off population, and because it can be broadly applied

to nearly all immune cell types, visualizing its gating across every individual lineage within Figure 1 would heavily clutter the main schematic and diminish the clarity of the primary gating framework.

Therefore, to maintain the illustrative clarity of the multi-organ framework while still demonstrating the panel's high-resolution capability, we chose to present its specific application in delineating the $CD117^+CD44^+$ subpopulation within the $CD127^+CD90^+$ innate lymphoid cell (ILC) compartment as a representative example (Fig 1).

We have also revised the Results section to explicitly articulate this broader, multi-purpose rationale for including CD44 in our multi-organ profiling strategy.

(1.10) Question about the Spectral Overlap of mScarlet

> *The panel adds mScarlet as an independent parameter, but its emission spectrum (peak ~570 nm) overlaps with PE (575 nm) and PE-eFluor 610 (610 nm). The authors show a spectral plot (Fig. S2) and claim compatibility, but no quantitative unmixing error analysis is provided for mScarlet. It would be preferable to use the sentence "mScarlet could be resolved as an additional parameter without unacceptable interference".*

We agree that the potential spectral overlap between mScarlet, PE, and PE-eFluor 610 is an important technical issue.

As detailed in Table S1, while these fluorochromes have overlapping profiles, they exhibit distinct peak signatures across different detectors. Specifically, PE, mScarlet, and PE-eFluor 610 peak at center wavelengths of 581 nm (B4), 598 nm (B5), and 615 nm (B6), respectively, which provides sufficient unique spectral information for effective unmixing on our 3-laser platform.

Following the reviewer's recommendation, we have updated the Methods section (2.5) to include the following statement: *"Based on this evaluation, mScarlet could be resolved as an additional parameter without unacceptable interference."*

(1.11) Question about the Inclusion of LIVE/DEAD Violet in Table 1

> *LIVE/DEAD violet is used in the protocol but not listed in Table 1. While it is not an antibody, including it in the fluorochrome table would improve completeness.*

We would like to respectfully clarify that LIVE/DEAD Violet is already included in Table 1. It is listed under the "Specificity" column as "Viability". We hope this clarification addresses the concern regarding the completeness of the protocol information.

(1.12) Question about the Exclusion of Male Mice and Potential Sex-Bias

> *All experiments used 6–8 week-old female mice. No male mice were included. This could introduce sex-bias, especially for immune responses and glucocorticoid production. Perhaps an explanation would be useful.*

Because immune responses and glucocorticoid production can differ between sexes, the exclusive use of female mice represents a limitation of the present study. The decision to use female mice was made to ensure experimental consistency and minimize confounding stress factors.

Unlike females, male mice often exhibit aggressive behavior and hierarchical fighting when co-housed. Since such social stress can significantly alter systemic glucocorticoid levels and immune profiles, we chose female mice to minimize these secondary variables during the inflammatory challenge.

We have updated the Discussion to acknowledge the exclusion of male mice as a limitation and noted that future studies incorporating both sexes will be necessary to generalize these findings.

(1.13) Question about the Classification of CD56^{+}CD90^{+} Neuronal-associated Cells

> *Calling CD56+CD90+ CD45- cells "neuronal cells" is biologically shaky. In mouse tissues, CD56/NCAM is not a clean neuron-specific marker, and CD90 is highly non-specific. Even within CD45- cells, this does not establish a neuronal identity. At best, they should call them:*
> *-neuronal-associated cells*
> *-NCAM/Thy1-positive non-immune cells*
> *-or remove that claim unless they validate with additional markers*
>
> *Two better marker examples they would need for a stronger neuronal claim:*
> *-Tuj1/βIII-tubulin*
> *-PGP9.5, NeuN, or other tissue-appropriate neural markers*
> *Right now, the manuscript oversells that population.*

We agree with the reviewer that CD56 and CD90 expressions within the CD45^{-} fraction are insufficient to definitively establish a neuronal identity without further marker validation.

As recommended, all instances previously referring to this population as "neuronal cells" have been changed to the more accurate and cautious term: "neuronal-associated cells."

(1.14) Question about the Specificity of Lineage Definitions Across Tissues

> *The paper claims broad multi-organ profiling, but some population definitions are too crude*

to justify the labels across all tissues.

Two examples:
-Monocytes = Ly6G-Ly6C+ across tissues. That is too simplistic in inflamed tissues and can capture mixed myeloid states.
-NK cells = NK1.1+. In some settings this risks contamination by NKT-like or other NK1.1+ populations unless lineage exclusion is clearer.

Another weak example:
-Macrophages are described within a CD11b-MHCII- compartment as F4/80+. That is not a biologically stable macrophage definition across tissues, because many tissue macrophages are CD11b+ and/or MHCII variable.

Characterizing multi-organ immune populations using a single panel introduces inherent trade-offs between high-throughput breadth and deep lineage discrimination.

- Regarding Monocytes and NK cells: Given the parameter constraints of a single multi-organ panel, we utilized well-established hallmark markers (Ly6C and NK1.1) to broadly capture these major lineages across diverse tissues. While we acknowledge that inflamed microenvironments can induce overlapping marker profiles (e.g., mixed myeloid states or NKT-like cells), further sub-fractionation would require highly specialized, tissue-specific markers, which exceeds the scope of our study.
- Regarding Macrophages: We agree that macrophage phenotypes vary significantly across different anatomical sites. We realize that our previous use of the phrase "unified gating framework" in the manuscript may have inadvertently implied the use of rigid, static quadrants across all tissues.

To clarify, while the overall hierarchical gating logic was unified, the precise gating boundaries were empirically adjusted for each tissue to accurately capture F4/80$^+$ populations. Figure 1 illustrates the spleen as a representative reference due to its diverse and comprehensive immune cell composition. Within this specific organ, we observed that the F4/80$^+$ macrophage population predominantly localized within the CD11b$^-$MHCII$^-$ compartment.

Accordingly, we have revised the Results section to explicitly clarify that while the overarching hierarchical framework is unified, the actual gating boundaries were flexibly and empirically adjusted for each organ in accordance with standard flow cytometry practices.

(1.15) Question about the Technical Terminology of Spectral Unmixing

This is a technical mistake. In spectral cytometry, the core process is spectral unmixing, not conventional compensation. The manuscript has a section called "Compensation" and a figure called "Compensation Correction." That is conceptually wrong or at least outdated

terminology for this context.

Two better alternatives would be to call
-“spectral unmixing”
-“unmixing adjustment” or “post-unmixing correction”

We completely agree with the reviewer’s correction regarding the technical terminology. As this study utilizes full spectrum cytometry, "spectral unmixing" is indeed the accurate term to describe the mathematical decomposition of the spectra.

Accordingly, we have systematically updated the manuscript, including the title of Methods section (2.6) and the title and legend of Figure S7. Specifically, "Compensation" and "Compensation Correction" have been replaced with "Spectral Unmixing" and "Unmixing Adjustment," respectively, to align with current field standards and accurately reflect the spectral analysis performed.

(2) Grammer Issues

(2.1) Question about the Imprecision of the Term Multiplexing

“Primarily due to the inherent difficulty of multiplexing.” This is vague sentence. The difficulty is more specifically attributed to spectral overlap and the scarcity of mouse-validated reagents; “multiplexing” alone is imprecise.

As suggested, we have revised the Abstract to specify that these challenges stem from spectral overlap and the constraints of reagent availability.

(2.2) Question about the Potential Competing Interest for a Specific Author

The authors declare “no competing interests exist” but immediately state that Y.L. is an employee of LK Bioscience Inc., a regional distributor for Cytek. This is a clear competing interest and should be declared as such.

We value the reviewer pointing out this issue. We agree that the professional affiliation of Y.L. should be formally disclosed as a potential competing interest to ensure full transparency. We have updated the Competing Interests declaration in the journal's submission system.

(2.3) Question about the Comparison between Human and Murine Study Methods

“Unlike human studies that rely primarily on peripheral blood, murine systems allow direct analysis of immune responses within organs” this sentence implies human studies cannot examine organs, which is untrue. It should be reworded to: “Unlike most human

> *immunological studies, which rely primarily on peripheral blood samples, murine systems readily allow..."*

To avoid any misinterpretation, we have updated the text as suggested to clarify that murine systems offer a more readily accessible approach to organ-specific analysis than typical human studies.

(2.4) Question about the Typo in Ly6C⁺ Monocyte Description

> *"Ly6C⁺ monocytes exhibiteda widespread increase", there is a missing space between "exhibited" and "a." This reads: "monocytes exhibited a widespread increase."*

We thank the reviewer for the meticulous reading of our manuscript. The missing space has been inserted as requested.

(2.5) Question about the Consistency of Abbreviations

> *"Consequently, researchers can readily apply sophisticated dimensionality reduction and clustering algorithms such as t-SNE (t-distributed Stochastic Neighbor Embedding), UMAP, and trajectory inference to dissect complex cellular networks using widely available cytometers." t-SNE is spelled out, but UMAP is not (Uniform Manifold Approximation and Projection is defined earlier in section 2.11). For consistency, either both should be expanded on first use or both abbreviated after prior definition.*

To ensure stylistic consistency within the sentence, we have opted to expand both terms. The revised text now provides the full names for both t-SNE and UMAP, ensuring a uniform and professional format for the reader.

(2.6) Question about the Consistency of Surface Marker Nomenclature

> *I saw*
> *-FceR1*
> *-FcεRI*
> *-Vio Bright B515 (FceRI)*
> *Pick one correct form and stick to it. For immunology, FcεRI is the clean version.*
> *Similarly, Ly-6C / Ly-6G*
> *-Ly6C / Ly6G*
> *Pick one and keep it consistent throughout. Also, "IL-7Ra" could be IL-7Rα.*

We apologize for the inconsistent terminology. We have standardized the nomenclature throughout the entire manuscript as follows:

- FcεRI
- Ly6C and Ly6G
- IL-7Rα

Reviewer #2

(1) Question about the Benchmarking and Unique Positioning of the Panel

> The development of a 27-color panel on a 3-laser system is technically valuable. However, the manuscript would benefit from clear benchmarking against existing OMIPs and high-parameter spectral panels. The authors should better articulate the unique advantages and positioning of their panel.

In response to this constructive feedback, we have thoroughly refined the introduction of our Results section to directly address these points. Specifically, we have articulated that while standardized multicolor spectral frameworks utilizing a 3-laser configuration are available for human immunophenotyping, a corresponding panel remains absent for murine models. Furthermore, we have explicitly contextualized our work against recently advanced murine spectral options like OMIP-105, noting that such panels strictly rely on 5-laser instrumentation and are limited to specific localized environments like the spleen and tumor.

By directly contrasting our design against these benchmarks, we effectively demonstrate how our 27-color configuration provides a practical, multi-organ alternative that maximizes the technical and biological value of widely accessible standard 3-laser systems.

(2) Question about the Quantitative Criteria for Fluorochrome Assignment

> The fluorochrome assignment strategy (e.g., brightness index, spreading error) is described, but quantitative criteria guiding these decisions are insufficiently detailed. Providing explicit thresholds or a structured framework would improve reproducibility.

We appreciate the reviewer's constructive feedback. We agree that detailing the panel design process improves the reproducibility of high-dimensional spectral cytometry.

To provide a clearer understanding of our strategy as recommended, we outline the structured framework that guided our panel design:

- First, we paired markers based on the inverse relationship between antigen density and fluorochrome brightness. Rather than relying on absolute numerical cutoffs, we utilized the relative brightness rankings provided by Cytek Cloud and paired them with relative antigen expression levels based on generally established biological knowledge.
- Second, we utilized the Spillover Spreading Matrix (SSM) to manage spreading risks. Fluorochromes with a higher propensity for spreading were strategically assigned to mutually exclusive markers to preserve the resolution of co-expressing populations.
- Third, these theoretical assignments were practically adjusted based on the commercial availability of specific antibody-fluorochrome conjugates, leading to our final panel.

We have updated the Methods section (2.4) to explicitly outline this structured approach, thereby providing clear guidance for reproducibility.

(3) Question about the Clarification of Mast Cell and Basophil Gating

The overall gating strategy is comprehensive and logically structured. However, the definition of mast cells and basophils requires clarification, as basophils are CD117- while mast cells are CD117+ (Fig. 1A). The current presentation may lead to misclassification.

We apologize for the confusion. The CD117 gating was actually performed during the analysis, but it was not clearly indicated. To address this issue, we have updated Figure 1A by adding clear arrows and larger labels to the respective gates. These annotations explicitly link the $CD117^{-}$ population to basophils and the $CD117^{+}$ population to mast cells, ensuring that the classification is immediately evident to the reader.

(4) Question about the Interpretation of mScarlet+Ly6C+ Monocytes

The identification of mScarlet+Ly6C+ monocytes across tissues is an interesting observation. However, this finding is not mechanistically explored and is currently overinterpreted as a biological conclusion rather than presented as an observational result. Additional validation or more cautious interpretation is recommended.

As the reviewer pointed out, we agree that while this phenomenon is interesting, it should be interpreted with caution.

To address the feedback, we have revised the text in the Results section to offer a more balanced interpretation. We have rephrased our descriptions to emphasize these findings as notable observations rather than definitive mechanistic conclusions. This ensures that further exploration is required to fully elucidate the underlying mechanisms.

Reference

Taves MD, Donahue KM, Bian J, Cam MC, Ashwell JD. Aire drives steroid hormone biosynthesis by medullary thymic epithelial cells. Sci Immunol. 2023;8(86):eabo7975.
