# Supplementary material for: An optimized three-laser 27-color spectral flow cytometry panel for multi-organ profiling in mice
Source: PLoS One. 2026 Jul 20;21(7):e0347810. doi: 10.1371/journal.pone.0347810 (PMC13384274; doi:10.1371/journal.pone.0347810)
Supplement: Attachment — Submitted filename: PLOSOne 27 color panel.docx [file pone.0347810.s014.docx]

Dear Editor,

Thank you for the opportunity to review the document. I have grouped the issues into two sections, Scientific and Grammatical. Should my review be scientifically more sensible, I would be happy to help you with critical reviews in future as well.

1. **Scientific / Methodological Issues**
   1.1. Statistical concerns

The study uses the Mann-Whitney U test throughout, which is appropriate for non-parametric comparisons. However, with only n= 4 to 5 mice per group (inferable from the dot plots in Figures 2 and 3), many comparisons yielding p=0.0571 are at the boundary of significance with this test's minimum achievable p-value for these sample sizes. The authors should clarify exact n per group in the methods, which is currently absent. The lack of multiple-testing correction (e.g., Benjamini-Hochberg) is not addressed despite dozens of simultaneous comparisons across tissues and cell types, raising the risk of false positives.

They use multiple tissue-by-cell-type comparisons and then report many p-values from Mann-Whitney tests, but I do not see any correction for multiple testing. That is a real weakness.

Two specific problems that I foresee

- many comparisons across tissues and populations inflate false-positive risk
- the sample size appears small from the figures, yet the claims are broad

At minimum I feel the authors should

- state exact n per group in figure legends or methods
- justify the statistical plan
- address multiplicity, or explicitly say the analysis is exploratory

Without that, some “significant” findings are less convincing.

1.2. Antibody panel design inconsistency

The authors state that CD90 is used to mark T cells, ILCs, and neuronal cells simultaneously, but this creates an inherent ambiguity in gating, especially in the lamina propria where all three populations coexist. The gating strategy relies on sequential marker exclusion to resolve this, but the manuscript does not explicitly address how CD90⁺ CD56⁺ neuronal cells are distinguished from CD90⁺ CD127⁺ ILCs when both reside in the CD45⁻ and CD45⁺ fractions respectively. This is a real interpretive concern that deserves direct acknowledgment.

1.3. mScarlet reporter interpretation

The authors identify Ly6C⁺ monocytes as "the most widespread and prominent glucocorticoid-producing immune population" based on elevated mScarlet expression. However, Cyp11b1 (11β-hydroxylase) encodes the enzyme for cortisol/corticosterone synthesis, but mScarlet reporter activity reflects Cyp11b1 promoter activation, not confirmed steroid secretion. The leap from reporter expression to "glucocorticoid production" is overstated without functional validation (e.g., intracellular steroid quantification, enzymatic activity assay). This should be softened to "Cyp11b1 reporter activity" throughout, or the distinction explicitly acknowledged.

It would be preferable to have orthogonal validation studies such as:

- steroid quantification from sorted populations
- CYP11B1 protein confirmation
- enzyme activity or pathway validation
- functional blockade or transcript-level confirmation in sorted cells

Two examples from the text:

- The abstract says they “identified a previously unrecognized, glucocorticoid-producing cell subset.”
- The Results/Discussion state that Ly6C+ monocytes are “the most widespread and prominent glucocorticoid-producing immune population.”
  1.4. Compensation bead avoidance rationale

In section 2.6, the authors avoid compensation beads citing "subtle spectral discrepancies." While the cited references (16, 17) support this concern for some fluorochromes, the blanket avoidance of beads for all 26–27 fluorochromes is not standard practice. Cell-based controls can themselves introduce variability (especially autofluorescence) depending on cell health and tissue of origin. This trade-off is not discussed, and the choice warrants more nuanced justification.

1.5. Autofluorescence model selection

The "Multiple AF Extraction (high AF)" model is applied uniformly across all tissues (Section 2.9), but the authors acknowledge that "subtle tissue-specific differences" exist. Applying a single high-AF model to tissues with variable autofluorescence (e.g., gut epithelium vs. peripheral blood) risks over-subtracting signal in low-AF samples. The rationale for this uniform approach over tissue-specific models is not adequately justified.

1.6. Poly(I:C) model — single time point limitation

All biological data are collected at exactly 24 hours post-injection. The authors describe this as capturing "dynamic shifts," but a single time point does not constitute a dynamic analysis. The word "dynamic" is used repeatedly (abstract, results, discussion) yet is not supported by the experimental design, which is inherently cross-sectional.

1.7. Missing negative control for mScarlet

The mScarlet signal in Ly6C⁺ monocytes is interpreted as biologically meaningful across all 8 tissues. However, Figure S9 (the only supplemental figure addressing Ly6C⁺ mScarlet expression in non-target tissues) shows only bone marrow and peripheral blood histograms, with very modest histogram shifts. The lack of a wild-type (non-reporter) mouse negative control stained with the full panel in every tissue is a notable gap . mScarlet gating relies entirely on the Cyp11b1-mScarlet reporter mice, and spectral unmixing artifacts from PE/PE-eFluor 610 (which flank mScarlet's emission; Fig. S2A) in non-reporter cells cannot be formally excluded without a true negative control.

1.8. Non-immune cell panel validation is limited

The non-immune gating strategy (Fig. 1B) is demonstrated only in the lamina propria. The paper claims the panel detects fibroblasts (CD140a⁺), endothelial cells (CD31⁺), epithelial cells (CD326⁺), and neuronal cells (CD56⁺ CD90⁺) across tissues, but validation in non-gut tissues for these populations is not shown. CD31 and CD56, for instance, are also expressed on immune/hematopoietic cells (NK cells express CD56; hematopoietic progenitors express CD31), and the reliance on a CD45⁻ gate alone may be insufficient to exclude all contaminating immune populations.

1.9. CD44 is included but not used in the gating strategy

CD44 (PE-Cy7) appears in Table 1 and S2 as part of the panel and is used only to gate ILCs (CD117⁺ CD44⁺), yet it is classified as a "secondary" activation marker (Table S3). Its contribution to the major gating framework shown in Figure 1 is marginal, and the rationale for its inclusion over other potentially more discriminating markers is not discussed.

1.10. Potential spectral conflict with mScarlet

The panel adds mScarlet as an independent parameter, but its emission spectrum (peak ~570 nm) overlaps with PE (575 nm) and PE‑eFluor 610 (610 nm). The authors show a spectral plot (Fig. S2) and claim compatibility, but no quantitative unmixing error analysis is provided for mScarlet. It would be preferable to use the sentence “mScarlet could be resolved as an additional parameter without unacceptable interference”

1.11. Missing viability dye in antibody table

LIVE/DEAD violet is used in the protocol but not listed in Table 1. While it is not an antibody, including it in the fluorochrome table would improve completeness.

1.12. Only female mice used

All experiments used 6–8 week‑old female mice. No male mice were included. This could introduce sex‑bias, especially for immune responses and glucocorticoid production. Perhaps an explanation would be useful.

1.13 “Neuronal cells” defined as CD56+ CD90+ is weak and likely misleading

Calling CD56+CD90+ CD45- cells “neuronal cells” is biologically shaky. In mouse tissues, CD56/NCAM is not a clean neuron-specific marker, and CD90 is highly non-specific. Even within CD45- cells, this does not establish a neuronal identity. At best, they should call them:

- neuronal-associated cells
- NCAM/Thy1-positive non-immune cells
- or remove that claim unless they validate with additional markers

Two better marker examples they would need for a stronger neuronal claim:

- Tuj1/βIII-tubulin
- PGP9.5, NeuN, or other tissue-appropriate neural markers

Right now, the manuscript oversells that population.

1.14 Several gated populations are too simplistically defined for cross-tissue claims

The paper claims broad multi-organ profiling, but some population definitions are too crude to justify the labels across all tissues.

Two examples:

- **Monocytes = Ly6G-Ly6C+** across tissues. That is too simplistic in inflamed tissues and can capture mixed myeloid states.
- **NK cells = NK1.1+**. In some settings this risks contamination by NKT-like or other NK1.1+ populations unless lineage exclusion is clearer.

Another weak example:

- **Macrophages** are described within a **CD11b-MHCII- compartment as F4/80+**. That is not a biologically stable macrophage definition across tissues, because many tissue macrophages are CD11b+ and/or MHCII variable.

1.15 They use “compensation” terminology where spectral flow requires “unmixing”

This is a technical mistake. In spectral cytometry, the core process is **spectral unmixing**, not conventional compensation. The manuscript has a section called **“Compensation”** and a figure called **“Compensation Correction.”** That is conceptually wrong or at least outdated terminology for this context.

Two better alternatives would be to call

- “spectral unmixing”
- “unmixing adjustment” or “post-unmixing correction”

2.0 **Grammer Issues**
    2.1 Abstract:

"Primarily due to the inherent difficulty of multiplexing". This is vague sentence. The difficulty is more specifically attributed to spectral overlap and the scarcity of mouse-validated reagents; "multiplexing" alone is imprecise.

2.2 Competing interest statement is contradictory

The authors declare “no competing interests exist” but immediately state that Y.L. is an employee of LK Bioscience Inc., a regional distributor for Cytek. This is a clear competing interest and should be declared as such.

2.3 Page 3, Introduction

"Unlike human studies that rely primarily on peripheral blood, murine systems allow direct analysis of immune responses within organs" this sentence implies human studies *cannot* examine organs, which is untrue. It should be reworded to: "Unlike most human immunological studies, which rely primarily on peripheral blood samples, murine systems readily allow..."

2.4 Page 14

"Ly6C⁺ monocytes exhibiteda widespread increase", there is a missing space between "exhibited" and "a." This reads: "monocytes exhibiteda widespread increase."

2.5 Page 16

"Consequently, researchers can readily apply sophisticated dimensionality reduction and clustering algorithms such as t-SNE (t-distributed Stochastic Neighbor Embedding), UMAP, and trajectory inference to dissect complex cellular networks using widely available cytometers." t-SNE is spelled out, but UMAP is not (Uniform Manifold Approximation and Projection is defined earlier in section 2.11). For consistency, either both should be expanded on first use or both abbreviated after prior definition.

2.6 Fc receptor naming is inconsistent

I saw

- FceR1
- FcεRI
- Vio Bright B515 (FceRI)

Pick one correct form and stick to it. For immunology, FcεRI is the clean version. Similarly, Ly-6C / Ly-6G

- Ly6C / Ly6G

Pick one and keep it consistent throughout. Also, “IL-7Ra” could be IL-7Rα
