# Supplementary material for: An optimized three-laser 27-color spectral flow cytometry panel for multi-organ profiling in mice
Source: PLoS One. 2026 Jul 20;21(7):e0347810. doi: 10.1371/journal.pone.0347810 (PMC13384274; doi:10.1371/journal.pone.0347810)
Supplement: Peer review history

# Peer review: Decision Letter 0

**DOI:** 10.1371/journal.pone.0347810.r001

**Authors:** Srinivasa Reddy Bonam

3 May 2026

PONE-D-26-16996

An optimized three-laser 27-color spectral flow cytometry panel for multi-organ profiling in mice

PLOS One

Dear Dr. Lim,

Thank you for submitting your manuscript to PLOS ONE. We have requested two reviewers to comment on your study. The reviewers' suggestions look very insightful. After careful consideration, we feel that it has merit but does not fully meet PLOS ONE’s publication criteria as it currently stands. Therefore, we invite you to submit a revised version of the manuscript that addresses the points raised during the review process.

Please ensure that your decision is justified on PLOS ONE’s publication criteria and not, for example, on novelty or perceived impact.

Please submit your revised manuscript by Jun 17 2026 11:59PM. If you will need more time than this to complete your revisions, please reply to this message or contact the journal office at plosone@plos.org. When you're ready to submit your revision, log on to https://www.editorialmanager.com/pone/ and select the 'Submissions Needing Revision' folder to locate your manuscript file.

Please include the following items when submitting your revised manuscript:

- A letter that responds to each point raised by the academic editor and reviewer(s). You should upload this letter as a separate file labeled 'Response to Reviewers'.
- A marked-up copy of your manuscript that highlights changes made to the original version. You should upload this as a separate file labeled 'Revised Manuscript with Track Changes'.
- An unmarked version of your revised paper without tracked changes. You should upload this as a separate file labeled 'Manuscript'.

If you would like to make changes to your financial disclosure, please include your updated statement in your cover letter. Guidelines for resubmitting your figure files are available below the reviewer comments at the end of this letter.

If applicable, we recommend that you deposit your laboratory protocols in protocols.io to enhance the reproducibility of your results. Protocols.io assigns your protocol its own identifier (DOI) so that it can be cited independently in the future. For instructions see: https://journals.plos.org/plosone/s/submission-guidelines#loc-laboratory-protocols. Additionally, PLOS ONE offers an option for publishing peer-reviewed Lab Protocol articles, which describe protocols hosted on protocols.io. Read more information on sharing protocols at https://plos.org/protocols?utm_medium=editorial-email&utm_source=authorletters&utm_campaign=protocols.

As the corresponding author, your ORCID iD is verified in the submission system and will appear in the published article. PLOS supports the use of ORCID, and we encourage all coauthors to register for an ORCID iD and use it as well. Please encourage your coauthors to verify their ORCID iD within the submission system before final acceptance, as unverified ORCID iDs will not appear in the published article. *Only*  the individual author can complete the verification step; PLOS staff *cannot*  verify ORCID iDs on behalf of authors.

We look forward to receiving your revised manuscript.

Kind regards,

Srinivasa Reddy Bonam

Academic Editor

PLOS One

Journal Requirements:

When submitting your revision, we need you to address these additional requirements.

1. Please ensure that your manuscript meets PLOS ONE's style requirements, including those for file naming. The PLOS ONE style templates can be found at

https://journals.plos.org/plosone/s/file?id=wjVg/PLOSOne_formatting_sample_main_body.pdf and

https://journals.plos.org/plosone/s/file?id=ba62/PLOSOne_formatting_sample_title_authors_affiliations.pdf

2. To comply with PLOS One submissions requirements, in your Methods section, please provide additional information regarding the experiments involving animals and ensure you have included details on (1) methods of sacrifice, (2) methods of anesthesia and/or analgesia, and (3) efforts to alleviate suffering

3. Thank you for stating the following in the Acknowledgments Section of your manuscript:

“This work was supported by the New Faculty Startup Fund (550-20230061), the Research Grant for Interdisciplinary Collaboration (550-20250081), the Research Institute for Veterinary Science, Seoul National University, and National Research Foundation (NRF), Ministry of Science, Information and Communication Technology (ICT) and Future Planning of Korea (RS-2021-NR057630, RS-2024-00335057 and RS-2024-00398705) (to J.L.).”

We note that you have provided additional information within the Acknowledgements Section that is not currently declared in your Funding Statement. Please note that funding information should not appear in the Acknowledgments section or other areas of your manuscript. We will only publish funding information present in the Funding Statement section of the online submission form.

Please remove any funding-related text from the manuscript and let us know how you would like to update your Funding Statement. Currently, your Funding Statement reads as follows:

“This work was supported by the New Faculty Startup Fund (550-20230061), the Research Grant for Interdisciplinary Collaboration (550-20250081), the Research Institute for Veterinary Science, Seoul National University, and National Research Foundation (NRF), Ministry of Science, Information and Communication Technology (ICT) and Future Planning of Korea (RS-2021-NR057630, RS-2024-00335057 and RS-2024-00398705) (to J.L.).”

Please include your amended statements within your cover letter; we will change the online submission form on your behalf.

4. Thank you for stating the following financial disclosure:

“This work was supported by the New Faculty Startup Fund (550-20230061), the Research Grant for Interdisciplinary Collaboration (550-20250081), the Research Institute for Veterinary Science, Seoul National University, and National Research Foundation (NRF), Ministry of Science, Information and Communication Technology (ICT) and Future Planning of Korea (RS-2021-NR057630, RS-2024-00335057 and RS-2024-00398705) (to J.L.).”

Please state what role the funders took in the study.  If the funders had no role, please state: "The funders had no role in study design, data collection and analysis, decision to publish, or preparation of the manuscript."

If this statement is not correct you must amend it as needed.

Please include this amended Role of Funder statement in your cover letter; we will change the online submission form on your behalf.

5. Thank you for stating the following in the Competing Interests section:

“All procedures were approved by the Institutional Animal Care and Use Committee (IACUC) of Seoul National University (SNU-240705-4-1) and conformed to institutional and national guidelines. Experiments were conducted and reported in accordance with ARRIVE guidelines.”

Please confirm that this does not alter your adherence to all PLOS ONE policies on sharing data and materials, by including the following statement: "This does not alter our adherence to  PLOS ONE policies on sharing data and materials.” (as detailed online in our guide for authors http://journals.plos.org/plosone/s/competing-interests).  If there are restrictions on sharing of data and/or materials, please state these. Please note that we cannot proceed with consideration of your article until this information has been declared.

Please include your updated Competing Interests statement in your cover letter; we will change the online submission form on your behalf.

6. Please note that your Data Availability Statement is currently missing a direct link to access each database. If your manuscript is accepted for publication, you will be asked to provide these details on a very short timeline. We therefore suggest that you provide this information now, though we will not hold up the peer review process if you are unable.

7. If the reviewer comments include a recommendation to cite specific previously published works, please review and evaluate these publications to determine whether they are relevant and should be cited. There is no requirement to cite these works unless the editor has indicated otherwise.

Reviewers' comments:

Reviewer's Responses to Questions

**Comments to the Author**

1. Is the manuscript technically sound, and do the data support the conclusions?

The manuscript must describe a technically sound piece of scientific research with data that supports the conclusions. Experiments must have been conducted rigorously, with appropriate controls, replication, and sample sizes. The conclusions must be drawn appropriately based on the data presented.

Reviewer #1: Yes

Reviewer #2: Partly

**********

2. Has the statistical analysis been performed appropriately and rigorously?

Reviewer #1: No

Reviewer #2: Yes

**********

3. Have the authors made all data underlying the findings in their manuscript fully available?

The PLOS Data policy requires authors to make all data underlying the findings described in their manuscript fully available without restriction, with rare exception (please refer to the Data Availability Statement in the manuscript PDF file). The data should be provided as part of the manuscript or its supporting information, or deposited to a public repository. For example, in addition to summary statistics, the data points behind means, medians and variance measures should be available. If there are restrictions on publicly sharing data—e.g. participant privacy or use of data from a third party—those must be specified.

Reviewer #1: Yes

Reviewer #2: Yes

**********

4. Is the manuscript presented in an intelligible fashion and written in standard English?

PLOS ONE does not copyedit accepted manuscripts, so the language in submitted articles must be clear, correct, and unambiguous. Any typographical or grammatical errors should be corrected at revision, so please note any specific errors here.

Reviewer #1: Yes

Reviewer #2: Yes

**********

5. Review Comments to the Author

Please use the space provided to explain your answers to the questions above. You may also include additional comments for the author, including concerns about dual publication, research ethics, or publication ethics. (Please upload your review as an attachment if it exceeds 20,000 characters)

Reviewer #1: The manuscript presents a valuable high‑dimensional flow cytometry panel, but few scientific issues require attention before publication – most notably the contradictory competing interest statement. The authors should address the unconventional ILC gating and provide additional validation for the neuronal marker CD56 in mouse tissues.Grammatical and formatting errors are mostly minor; they can be corrected during proofreading.

Reviewer #2: In the current manuscript the authors describe the development and optimization of a 27-color spectral flow cytometry panel using a 3-laser configuration for multi-organ immune profiling in mice. The study is technically relevant and addresses a practical gap in murine high-dimensional cytometry using widely accessible instrumentation. However, several concerns should be addressed prior to consideration for publication:

• The development of a 27-color panel on a 3-laser system is technically valuable. However, the manuscript would benefit from clear benchmarking against existing OMIPs and high-parameter spectral panels. The authors should better articulate the unique advantages and positioning of their panel.

• The fluorochrome assignment strategy (e.g., brightness index, spreading error) is described, but quantitative criteria guiding these decisions are insufficiently detailed. Providing explicit thresholds or a structured framework would improve reproducibility.

• The overall gating strategy is comprehensive and logically structured. However, the definition of mast cells and basophils requires clarification, as basophils are CD117- while mast cells are CD117+ (Fig. 1A). The current presentation may lead to misclassification.

• The identification of mScarlet+Ly6C+ monocytes across tissues is an interesting observation. However, this finding is not mechanistically explored and is currently overinterpreted as a biological conclusion rather than presented as an observational result. Additional validation or more cautious interpretation is recommended.

**********

6. PLOS authors have the option to publish the peer review history of their article (what does this mean?). If published, this will include your full peer review and any attached files.

If you choose “no”, your identity will remain anonymous but your review may still be made public.

**Do you want your identity to be public for this peer review?**  For information about this choice, including consent withdrawal, please see our Privacy Policy.

Reviewer #1: **Yes:**  Natarajan Badri Narayan

Reviewer #2: No

**********

[NOTE: If reviewer comments were submitted as an attachment file, they will be attached to this email and accessible via the submission site. Please log into your account, locate the manuscript record, and check for the action link "View Attachments". If this link does not appear, there are no attachment files.]

To ensure your figures meet our technical requirements, please review our figure guidelines: https://journals.plos.org/plosone/s/figures

You may also use PLOS’s free figure tool, NAAS, to help you prepare publication quality figures: https://journals.plos.org/plosone/s/figures#loc-tools-for-figure-preparation.

NAAS will assess whether your figures meet our technical requirements by comparing each figure against our figure specifications.

Attachment Submitted filename: PLOSOne 27 color panel.docx

# Author comment: Author response to Decision Letter 1

**DOI:** 10.1371/journal.pone.0347810.r002

10 Jun 2026

Please find the attached Response to Reviewers.

Attachment Submitted filename: Response to Reviewers.pdf

# Peer review: Decision Letter 1

**DOI:** 10.1371/journal.pone.0347810.r003

**Authors:** Srinivasa Reddy Bonam

30 Jun 2026

An optimized three-laser 27-color spectral flow cytometry panel for multi-organ profiling in mice

PONE-D-26-16996R1

Dear Dr. Jaechul Lim,

We’re pleased to inform you that your manuscript has been judged scientifically suitable for publication and will be formally accepted for publication once it meets all outstanding technical requirements.

Within one week, you’ll receive an e-mail detailing the required amendments. When these have been addressed, you’ll receive a formal acceptance letter and your manuscript will be scheduled for publication.

An invoice will be generated when your article is formally accepted. Please note, if your institution has a publishing partnership with PLOS and your article meets the relevant criteria, all or part of your publication costs will be covered. Please make sure your user information is up-to-date by logging into Editorial Manager at Editorial Manager® and clicking the ‘Update My Information' link at the top of the page. For questions related to billing, please contact billing support.

If your institution or institutions have a press office, please notify them about your upcoming paper to help maximize its impact. If they’ll be preparing press materials, please inform our press team as soon as possible -- no later than 48 hours after receiving the formal acceptance. Your manuscript will remain under strict press embargo until 2 pm Eastern Time on the date of publication. For more information, please contact onepress@plos.org.

Kind regards,

Srinivasa Reddy Bonam

Academic Editor

PLOS One

Additional Editor Comments (optional):

Reviewers' comments:

Reviewer's Responses to Questions

**Comments to the Author**

1. If the authors have adequately addressed your comments raised in a previous round of review and you feel that this manuscript is now acceptable for publication, you may indicate that here to bypass the “Comments to the Author” section, enter your conflict of interest statement in the “Confidential to Editor” section, and submit your "Accept" recommendation.

Reviewer #1: All comments have been addressed

Reviewer #2: All comments have been addressed

**********

2. Is the manuscript technically sound, and do the data support the conclusions?

The manuscript must describe a technically sound piece of scientific research with data that supports the conclusions. Experiments must have been conducted rigorously, with appropriate controls, replication, and sample sizes. The conclusions must be drawn appropriately based on the data presented.

Reviewer #1: Yes

Reviewer #2: Yes

**********

3. Has the statistical analysis been performed appropriately and rigorously?

Reviewer #1: Yes

Reviewer #2: I Don't Know

**********

4. Have the authors made all data underlying the findings in their manuscript fully available?

The PLOS Data policy requires authors to make all data underlying the findings described in their manuscript fully available without restriction, with rare exception (please refer to the Data Availability Statement in the manuscript PDF file). The data should be provided as part of the manuscript or its supporting information, or deposited to a public repository. For example, in addition to summary statistics, the data points behind means, medians and variance measures should be available. If there are restrictions on publicly sharing data—e.g. participant privacy or use of data from a third party—those must be specified.

Reviewer #1: Yes

Reviewer #2: Yes

**********

5. Is the manuscript presented in an intelligible fashion and written in standard English?

PLOS ONE does not copyedit accepted manuscripts, so the language in submitted articles must be clear, correct, and unambiguous. Any typographical or grammatical errors should be corrected at revision, so please note any specific errors here.

Reviewer #1: Yes

Reviewer #2: Yes

**********

6. Review Comments to the Author

Please use the space provided to explain your answers to the questions above. You may also include additional comments for the author, including concerns about dual publication, research ethics, or publication ethics. (Please upload your review as an attachment if it exceeds 20,000 characters)

Reviewer #1: This is a methodologically sound, well-executed, and genuinely useful technical resource paper, and I would recommend acceptance after minor revisions.

Core scientific contribution is real and addresses a genuine gap:

The central claim holds up: there is a documented scarcity of high-dimensional murine panels that work on standard 3-laser instruments, and the existing alternatives (OMIP-104, OMIP-105, and similar) either require 5-laser systems or are restricted to single tissues (spleen, tumor). A 27-color panel that runs on the much more widely available 3-laser Aurora and spans eight tissues simultaneously is a meaningful and practically valuable advance for the field. This isn't an incremental panel tweak — it solves an accessibility problem that many labs without 4–5 laser instruments genuinely face.

Panel design methodology is rigorous and well-documented:

The fluorochrome assignment logic (antigen density vs. brightness inversely paired, SSM-guided redistribution of high-spread combinations, iterative practical adjustment for reagent availability) follows established best practice (OMIP-069 framework) and is clearly described with enough granularity to be reproducible — exactly what a methods-focused OMIP-style paper should deliver. The titration strategy, FMO control use, and tissue-specific autofluorescence extraction are all appropriately justified and documented in the supplementary figures.

The authors responded to peer review in good faith and substantively

This is worth weighing heavily in a positive recommendation. Across both reviewers, the authors:

Added a full FDR-corrected statistical reanalysis rather than dismissing the multiplicity concern

Walked back overstated language ("glucocorticoid-producing" → "Cyp11b1-expressing"; "dynamic" → "acute") rather than defending indefensible claims

Disclosed the COI properly once flagged, rather than minimizing it

Added new validation data (Fig R1–R3) directly addressing specificity and cross-tissue gating concerns

Engaged with even minor terminology critiques (spectral unmixing vs. compensation, FcRI standardization) with full corrections

This is the behavior we want to see in a revision cycle, substantive engagement rather than perfunctory point-by-point deflection.

Appropriate epistemic humility in the Discussion

The limitations section is honest and complete: it explicitly flags the lack of intracellular/functional readouts, the female-only mouse cohort, the coarse-grained lineage definitions, and residual spectral spreading. This is exactly the kind of self-aware limitations section that strengthens rather than weakens a methods paper — it tells future users of the panel precisely where its boundaries are.

Biological validation (poly(I:C) model) is a credible proof-of-concept, not overreach once corrected

Using poly(I:C) as a systemic inflammatory challenge to demonstrate the panel can resolve coordinated multi-tissue immune remodeling is an appropriate and interpretable validation strategy. The finding of compartment-specific T cell dynamics (peripheral reduction vs. thymic stability) and the discovery of Ly6C⁺ monocyte Cyp11b1-reporter activity are interesting observations that, now properly hedged as reporter-level findings rather than confirmed steroidogenesis, are reasonable and additive to the literature.

Reviewer #2: (No Response)

**********

7. PLOS authors have the option to publish the peer review history of their article (what does this mean?). If published, this will include your full peer review and any attached files.

If you choose “no”, your identity will remain anonymous but your review may still be made public.

**Do you want your identity to be public for this peer review?**  For information about this choice, including consent withdrawal, please see our Privacy Policy.

Reviewer #1: **Yes:**  Natarajan Badri Narayan

Reviewer #2: No

**********

# Editor report: Acceptance letter

**DOI:** 10.1371/journal.pone.0347810.r004

**Authors:** Srinivasa Reddy Bonam

PONE-D-26-16996R1

PLOS One

Dear Dr. Lim,

I'm pleased to inform you that your manuscript has been deemed suitable for publication in PLOS One. Congratulations! Your manuscript is now being handed over to our production team.

At this stage, our production department will prepare your paper for publication. This includes ensuring the following:

* All references, tables, and figures are properly cited

* All relevant supporting information is included in the manuscript submission,

* There are no issues that prevent the paper from being properly typeset

You will receive further instructions from the production team, including instructions on how to review your proof when it is ready. Please keep in mind that we are working through a large volume of accepted articles, so please give us a few days to review your paper and let you know the next and final steps.

Lastly, if your institution or institutions have a press office, please let them know about your upcoming paper now to help maximize its impact. If they'll be preparing press materials, please inform our press team within the next 48 hours. Your manuscript will remain under strict press embargo until 2 pm Eastern Time on the date of publication. For more information, please contact onepress@plos.org.

You will receive an invoice from PLOS for your publication fee after your manuscript has reached the completed accept phase. If you receive an email requesting payment before acceptance or for any other service, this may be a phishing scheme. Learn how to identify phishing emails and protect your accounts at https://explore.plos.org/phishing.

If we can help with anything else, please email us at customercare@plos.org.

Thank you for submitting your work to PLOS ONE and supporting open access.

Kind regards,

PLOS ONE Editorial Office Staff

on behalf of

Dr. Srinivasa Reddy Bonam

Academic Editor

PLOS One
